# Somatic, germline and sex hierarchy regulated gene expression during Drosophila metamorphosis

**DOI:** 10.1186/1471-2164-10-80

**Published:** 2009-02-13

**Authors:** Matthew S Lebo, Laura E Sanders, Fengzhu Sun, Michelle N Arbeitman

**Affiliations:** 1 Section of Molecular and Computational Biology, Department of Biological Sciences, University of Southern California, Los Angeles, California 90089, USA; 2 Section of Neurobiology, Department of Biological Sciences, University of Southern California, Los Angeles, California 90089, USA

## Abstract

**Background:**

*Drosophila melanogaster *undergoes a complete metamorphosis, during which time the larval male and female forms transition into sexually dimorphic, reproductive adult forms. To understand this complex morphogenetic process at a molecular-genetic level, whole genome microarray analyses were performed.

**Results:**

The temporal gene expression patterns during metamorphosis were determined for all predicted genes, in both somatic and germline tissues of males and females separately. Temporal changes in transcript abundance for genes of known functions were found to correlate with known developmental processes that occur during metamorphosis. We find that large numbers of genes are sex-differentially expressed in both male and female germline tissues, and relatively few are sex-differentially expressed in somatic tissues. The majority of genes with somatic, sex-differential expression were found to be expressed in a stage-specific manner, suggesting that they mediate discrete developmental events. The *Sex-lethal *paralog, *CG3056*, displays somatic, male-biased expression at several time points in metamorphosis. Gene expression downstream of the somatic, sex determination genes *transformer *and *doublesex (dsx) *was examined in two-day old pupae, which allowed for the identification of genes regulated as a consequence of the sex determination hierarchy. These include the homeotic gene *abdominal A*, which is more highly expressed in females as compared to males, as a consequence of *dsx*. For most genes regulated downstream of *dsx *during pupal development, the mode of regulation is distinct from that observed for the well-studied direct targets of DSX, *Yolk protein 1 *and *2*.

**Conclusion:**

The data and analyses presented here provide a comprehensive assessment of gene expression during metamorphosis in each sex, in both somatic and germline tissues. Many of the genes that underlie critical developmental processes during metamorphosis, including sex-specific processes, have been identified. These results provide a framework for further functional studies on the regulation of sex-specific development.

## Background

In *Drosophila melanogaster*, metamorphosis is the period in development when the male and female larval forms, which display little morphological sexual dimorphism, are transformed into the reproductive male and female adult forms, which display large differences between the sexes. This complete transformation is the result of several processes [reviewed in [1]]: the degeneration of somatic larval structures; the generation of adult structures from cells that are found within the larva (imaginal discs, imaginal rings and histoblast nests); remodeling, death and neurogenesis of the cells in the larval nervous system; and the development of the adult gonads through interactions of both germline and the somatic tissues. Identifying the genes that underlie and orchestrate this transformation, and understanding how sex-specific gene regulation is integrated into these processes will provide insight into how large-scale changes in morphology are directed at a molecular-genetic level.

Metamorphosis initiates at the end of the third larval instar by a pulse of the steroid hormone ecdysone [reviewed in [1]]. In response to this pulse of ecdysone, the larva ceases movement and initiates pre-pupal development. Progression through the subsequent pupal stages is mediated by an additional pre-pupal pulse of ecdysone that triggers pupal formation, and finally by a large pulse of ecdysone that triggers adult development [reviewed in [1]]. While much is known about the morphological changes that occur during metamorphosis [reviewed in [2]], less is known about the gene expression changes that occur specifically in somatic and germline tissues that underlie these changes, and how sex-specific regulation of gene expression is incorporated into the developmental pathways.

**Figure 1:**
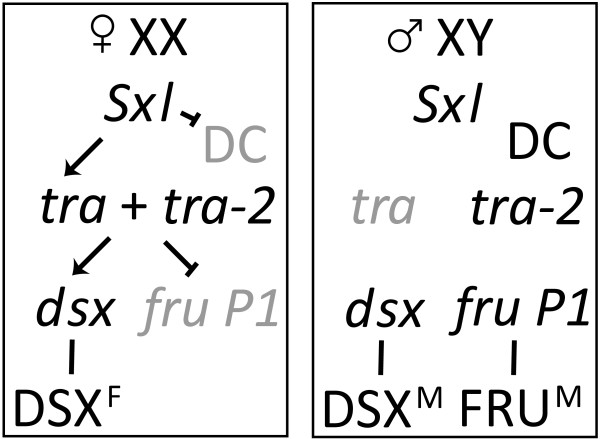
**The *Drosophila melanogaster *sex determination hierarchy**. The Drosophila sex determination hierarchy consists of a cascade of sex-specific alternatively spliced pre-mRNAs culminating in the production of sex-specific transcription factors encoded by *doublesex *(*dsx*) and *fruitless P1 *(*fru P1*). The primary determinate of sex is the X chromosome to autosomal chromosome (A) ratio. In females (X:A = 1), Sex Lethal (SXL) is produced and regulates the splicing of the pre-mRNA of *transformer *(*tra*), resulting in the production of TRA. TRA acts in conjunction with constitutively produced TRA-2, and regulates the alternative splicing of the pre-mRNAs of *dsx *and *fru P1*, leading to the production of the female-specific protein DSX^F^. In males (X:A = 0.5), SXL is not produced and *dsx *and *fru P1 *pre-mRNAs undergo default splicing, resulting in the production of the male sex-specific proteins DSX^M ^and FRU^M^. In addition, in females SXL represses dosage compensation, the process by which transcription of genes on the single X chromosome in males is up-regulated to roughly equal that of the two X chromosomes in females.

Insight into somatic, sexual development in Drosophila is provided by the study of the sex determination hierarchy (see Figure 1), a genetic regulatory hierarchy consisting of a sexually dimorphic pre-mRNA splicing cascade that terminates with the production of sex-specific transcription factors encoded by *doublesex *(*dsx*) and *fruitless *(*fru*) [reviewed in [3]]. *dsx *controls all morphological differences between the sexes [reviewed in [3]], whereas *fru *is necessary for nearly all aspects of male courtship behaviors [reviewed in [4]]. While much is known about adult, sex-specific phenotypes caused by mutations in *dsx *and *fru *[5,6], how *dsx *and *fru *direct sex-specific development at the level of gene expression during metamorphosis is still an open question.

Previous studies have examined gene expression during metamorphosis, though the studies were limited in the number of genes assayed and in the fact that they did not distinguish between gene expression in somatic and germline tissues [7,8]. In this study, a microarray-based approach was used to examine expression from all predicted genes, in both wild type flies and flies that lack germline tissues, during metamorphosis. Additionally, both the role of *dsx *in establishing somatic sex differences in gene transcript levels and the modes of how *dsx *regulates gene expression were determined.

## Results and discussion

Here, genes that underlie developmental changes that occur during metamorphosis were identified by assaying gene expression in male and female wild type animals and animals that lack germline tissues, using a two-color, glass-slide microarray approach (see Methods; [9]). The wild type animals are the Canton S (CS) strain and the animals that lack germline tissues are the progeny of female flies homozygous for the maternal-effect, recessive mutation *tudor *(*tud*), hereafter referred to as *tud *progeny [10]. Experimental samples were compared to a common reference sample consisting of RNA derived from male and female CS pupae collected from all stages of metamorphosis; this approach facilitated comparisons across all the experiments (see Table 1 for experimental design). Gene expression was assayed at five time points in animals collected every 24 hours, ranging from 0 hours after puparium formation (APF; 0 hour APF is the white pre-pupal stage) to 96 hour APF (pharate adults).

**Table 1:** Microarray experimental design for time course and sex hierarchy experiments.

**I. Time course experiment**				
**Probe 1**	**Probe 2**	**Replicates**	**Time points (hr APF)**	
Wild type female (CS)	Reference	3	0, 24, 48, 72, 96	
Wild type male (CS)	Reference	3	0, 24, 48, 72, 96	
*tud *progeny female	Reference	3	0, 24, 48, 72, 96	
*tud *progeny male	Reference	3	0, 24, 48, 72, 96	

II. Sex determination hierarchy experiment*				

**Probe 1**	**Probe 2**	**Replicates**	**Time point (hr APF)**	**# of Genes**^†^
Wild type female (CS)	Wild type male (CS)	4	48	7972
Wild type female (Berlin)	Wild type male (Berlin)	4	48	
*tud *progeny female	*tud *progeny male	4	48	420
Wild type female (CS)	XX *tra *pseudomales	4	48	95
Wild type female (CS)	XX *dsx *^*D *^pseudomales	4	48	173**
Wild type female (CS)	XX *dsx *intersexuals	4	48	154
Wild type male (CS)	XY *dsx *intersexuals	4	48	155

Probes, numbers of replicates, and the time points assayed are listed for each experiment. Chromosomal sex is indicated by XX or XY. * Sex determination hierarchy experiments were all performed with a dye-swap design (see Methods). ^† ^The number of genes in each experiment that is significantly differentially expressed. ** The DSX set was defined with an additional statistical test to eliminate potential false-negatives resulting from exclusion in the TRA set. Therefore, a gene's inclusion in the DSX set is not necessarily contingent on its presence in the TRA set. For a description of the definition of the DSX set, see Results and Discussion and Methods.

Additionally, somatic, sex differences in transcript abundance for genes regulated downstream of *dsx *(Figure 1) were determined at a mid-pupal stage (48 hour APF). Microarray comparisons using RNA from the following genotypes were performed: wild type males and females from two different strains (CS and Berlin), male and female *tud *progeny, wild type females and *tra *pseudomales, and wild type females and *dsx*^*D *^pseudomales. *tra *and *dsx*^*D *^pseudomales are chromosomally XX animals that produce DSX^M^, the male-specific isoform of DSX, and as a result look phenotypically similar to wild type males [11,12]. The analyses of two distinct mutant genotypes that produce DSX^M ^in a chromosomally XX background facilitated the identification of genes that are sex-differentially expressed downstream of DSX, as opposed to differences in sex-chromosome content, and together with the analyses of two different wild type strains, reduced the identification of genes for which differential expression is due to differences in strain, or genes acting only upstream of *dsx *and/or *tra *in the sex hierarchy. Additionally, gene expression was compared between intersexual male and female flies that do not produce DSX (*dsx *null; *dsx*^*d*+*r*3^/*dsx*^*m*+*r*15 ^[5]) and wild type males and females, respectively, to examine the modes of *dsx*-regulated gene expression (see Table 1).

### Time course experiment: gene expression during metamorphosis

The expression data from both wild type and *tud *progeny males and females was first analyzed to identify genes expressed in somatic and germline tissues during metamorphosis. Here, 8,482 and 9,725 genes of the 13,820 genes examined had expression data in the *tud *and wild type experiments, respectively, demonstrating that ~70% (9,725 of 13,820 genes in wild type flies) of the predicted Drosophila genes are expressed during metamorphosis (see Methods for details). This also suggests that approximately 1,200 additional genes are expressed during metamorphosis due to the presence of the germline in wild type males and females. Our previous study examining gene expression in wild type flies found a larger percentage of genes (~94% of genes represented on arrays) expressed during metamorphosis (3,784/4,028 genes; [7]). Our previous study employed a cDNA microarray platform representing about one-third of the genes in *Drosophila*. As such, it was biased for genes with high expression levels, which might account for the differences in the two studies [7].

### Somatic sex-differential gene expression during metamorphosis

To identify genes whose transcript abundances differ between the two sexes in somatic tissues through metamorphosis, the *tud *progeny gene expression data was analyzed using F-statistics, conducted using LIMMA contrasts with sex and time as independent factors (see Methods and Additional file 1, for details). For the F-test analyses, lists of *P *values were converted to *q *values, an estimate of the false discovery rate [13]. Two-hundred-fifty-eight genes were identified with significant somatic, sex-differential expression (*q *< 0.15 for sex or sex-time interaction term; see Methods for details; Additional files 2 and 3). Similar numbers of genes were identified with male- and female-biased expression (124 and 134, respectively). Overall, the percentage of genes with somatic, sex-differential expression during pupal stages (1.9%) is similar to the number of genes displaying somatic, sex-differential expression at adult stages (1.7% of genes [68/4028] in [7], 2.5% genes [301/11893] in [14], and 1.4% of genes [167/11893] in [15]). In contrast, thousands of genes show sex-differences in transcript levels in the male and female germline tissues, at both pupal and adult stages (see germline section below; [7,14]).

**Figure 2:**
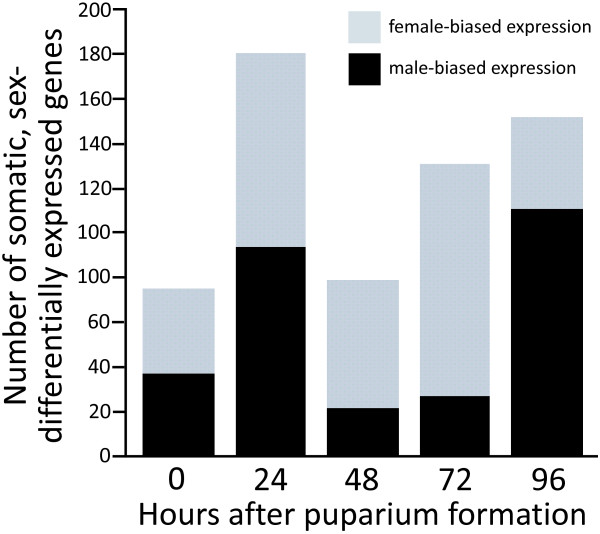
**The number of genes with somatic sex-differential transcript levels differs across metamorphosis**. The abscissa indicates the five time points during metamorphosis examined (0, 24, 48, 72, and 96 hour APF). The ordinate indicates the number of somatic, sex-differentially expressed genes, as identified by F-tests, *q *< 0.15, for sex or sex-time interaction terms, and a *t*-test of the means (*q *< 0.15), on the gene expression data of male and female *tud *progeny. Genes with female- and male-biased expression are shown in grey and black, respectively.

For the 258 genes with somatic, sex-biased expression, moderated-*t*-tests were performed [16], comparing gene expression in *tud *progeny males and females to determine at which stage the gene displays somatic, sex-differential expression (Table 2). The five time points (0, 24, 48, 72 and 96 hour APF) do not have large differences in the numbers of genes with somatic, sex-biased transcript levels (*q *< 0.15; 75, 181, 79, 131, and 152 genes, respectively; Figure 2), with the data from the 24 hour time point containing the largest number of genes.

**Table 2:** Sex differentially expressed genes in somatic tissues during metamorphosis.

**Hr APF**	**Sex-bias**	**Gene Name**	CG # *	**Ch **^†^	**FC **^‡^	**Functional Annotation**^ξ^
0 hr	Male	*RNA on the X 1*	CR32777	X	24.1	dosage compensation
		*CG1441*	CG1441	2R	2.5	catalytic activity; binding
						
	Female	*CG4500*	CG4500	2L	2.7	mesoderm development
		*Larval serum protein 1 α*	CG2559	X	2.3	nutrient reservoir activity; oxygen transporter
		*Transferrin 1*	CG6186	X	2.0	iron ion transport; defense response
						
24 hr	Male	*RNA on the X 1*	CR32777	X	150.6	dosage compensation
		*dusky*	CG9355	X	16.1	wing morphogenesis
		*CG1368*	CG1368	X	5.0	structural constituent of chorion
		*CG5823*	CG5823	3R	4.9	ubiquitin-protein ligase activity
		*CG31948*	CG31948	2L	3.8	protein modification process
		*CG4691*	CG4691	2L	3.6	unknown
		*Cyto dynein light chain 2*	CG5450	2L	3.5	microtubule motor activity
		*CG11350*	CG11350	3L	3.2	unknown
		*CG6372*	CG6372	3L	3.0	mushroom body development; proteolysis
		*CG10383*	CG10383	2L	2.6	binding
		*Moesin*	CG10701	X	2.2	cytoskeletal protein binding; actin binding
		*CG2082*	CG2082	3R	2.2	unknown
		*CG11905*	CG11905	3L	2.2	hydrolase activity
		*Cuticular protein 12A*	CG15757	X	2.0	structural constituent of cuticle
		*Mov34*	CG3416	2R	2.0	endopeptidase activity; cell proliferation
						
	Female	*CG2930*	CG2930	X	4.1	oligopeptide transport
		*CG10650*	CG10650	2L	3.2	oxidoreductase activity
		*CG12290*	CG12290	3R	2.4	G-protein coupled receptor activity
		*CG14540*	CG14540	3R	2.4	unknown
		*CG16997*	CG16997	2L	2.3	serine-type endopeptidase activity
		*CG7045*	CG7045	3R	2.3	regulation of transcription; DNA binding
		*CG9634*	CG9634	X	2.3	metalloendopeptidase activity
		*white*	CG2759	X	2.3	transmembrane receptor activity; eye pigment
		*26–29 kD-proteinase*	CG8947	3L	2.3	cathepsin K activity; proteolysis
		*CG32695*	CG32695	X	2.3	unknown
		*ion transport peptide*	CG13586	2R	2.2	neuropeptide hormone activity
		*CG15312*	CG15312	X	2.1	unknown
		*CG15369*	CG15369	X	2.1	cysteine protease inhibitor activity
		*CG16865*	CG16865	2L	2.0	unknown
						
48 hr	Male	*RNA on the X 1*	CR32777	X	232.8	dosage compensation
		*CG31988*	CG31988	2L	5.2	zinc ion binding
		*bangles and beads*	CG7088	X	2.3	gliogenesis
		*CG1368*	CG1368	X	2.3	structural constituent of chorion
		*CG9914*	CG9914	X	2.0	fatty acid metabolic process
		*CG31948*	CG31948	2L	2.0	protein modification process
						
	Female	*Cuticular protein 12A*	CG15757	X	5.0	structural constituent of cuticle
		*Hs protein cognate 2*	CG7756	3R	5.0	unfolded protein binding; ATP binding
		*CG6416*	CG6416	3L	3.6	mesoderm development; protein binding
		*CG1441*	CG1441	2R	3.5	catalytic activity; binding
		*CG12523*	CG12523	3L	3.1	unknown
		*Mec2*	CG7635	X	2.8	unknown
		*CG32982*	CG32982	2L	2.8	unknown
		*CG8745*	CG8745	3L	2.6	arginine catabolic process to glutamate
		*gonadal*	CG33756	3L	2.5	spermatogenesis; oogenesis
		*CG12009*	CG12009	3L	2.5	chitin binding; chitin metabolism
		*Larval serum protein 1 α*	CG2559	X	2.4	nutrient reservoir activity; oxygen transporter
		*CG7367*	CG7367	2L	2.3	phagocytosis; lipid metabolic process
		*CG15369*	CG15369	X	2.2	cysteine protease inhibitor activity
		*CG31901*	CG31901	2L	2.2	unknown
		*CG13360*	CG13360	X	2.1	unknown
		*CG10589*	CG10589	3L	2.1	unknown
						
72 hr	Male	*RNA on the X 1*	CR32777	X	147.6	dosage compensation
		*CG7191*	CG7191	2L	2.1	unknown
		*Verprolin 1*	CG13503		2.1	actin filament organization
						
	Female	*CG11591*	CG11591	3L	6.2	unknown
		*CG31948*	CG31948	2L	5.5	protein modification process
		*CG32690*	CG32690	X	5.1	unknown
		*CG7738*	CG7738		4.0	
		*CG10589*	CG10589	3L	3.8	unknown
		*CG9975*	CG9975	2R	3.7	unknown
		*Cuticular protein 12A*	CG15757	X	3.7	structural constituent of cuticle
		*CG31639*	CG31639	2L	3.3	unknown
		*CG4691*	CG4691	2L	3.1	unknown
		*Jonah 25Biii*	CG8871	2L	3.1	elastase activity; serine-type endopeptidase
		*CG7322*	CG7322	X	3.0	oxidoreductase activity
		*Lysozyme B*	CG1179	3L	2.9	lysozyme activity
		*CG17118*	CG17118	2L	2.6	dendrite morphogenesis
		*CG8813*	CG8813	2L	2.6	unknown
		*CG13360*	CG13360	X	2.4	unknown
		*CG10809*	CG10809	3L	2.4	unknown
		*CG5446*	CG5446	2L	2.4	unknown
		*CG14540*	CG14540	3R	2.3	unknown
		*Peroxiredoxin 2540*	CG11765	2R	2.3	glutathione peroxidase activity
		*CG31901*	CG31901	2L	2.2	unknown
		*abdominal A*	CG10325	3R	2.2	transcription factor; organismal development
		*CG15369*	CG15369	X	2.1	cysteine protease inhibitor activity
		*Larval serum protein 1 α*	CG2559	X	2.1	nutrient reservoir activity; oxygen transporter
		*CG12250*	CG12250	3R	2.1	unknown
		*CG17244*	CG17244	3R	2.0	unknown
		*CG1998*	CG1998	X	2.0	C-4 methylsterol oxidase activity
						
96 hr	Male	*RNA on the X 1*	CR32777	X	325.5	dosage compensation
		*CG31988*	CG31988	2L	7.8	zinc ion binding
		*CG6372*	CG6372	3L	5.7	mushroom body development; proteolysis
		*Cyt. dynein light chain 2*	CG5450	2L	4.1	microtubule motor activity
		*charlatan*	CG11798	2R	4.1	nervous system development; transcription
		*CG17829*	CG17829	X	3.8	zinc ion binding
		*CG1774*	CG1774	3R	3.7	catalytic activity; nucleotide binding
		*CG31029*	CG31029	3R	3.6	unknown
		*Syntaxin Interact. Prot. 2*	CG13164	2R	3.5	unknown
		*Lysozyme B*	CG1179	3L	3.3	lysozyme activity
		*CG4691*	CG4691	2L	3.3	unknown
		*carmine*	CG3035	X	3.1	neurotransmitter secretion; synaptic vesicle
		*CG4300*	CG4300	3L	2.7	spermidine synthase activity
		*peste*	CG7228	2L	2.7	scavenger receptor activity
		*dacapo*	CG1772	2R	2.7	cyclin-dependent protein kinase inhibitor
		*CG8813*	CG8813	2L	2.6	unknown
		*Ribosomal protein S5b*	CG7104	3R	2.4	structural constituent of ribosome
		*CG12237*	CG12237	X	2.4	phosphoric monoester hydrolase activity
		*CG7738*	CG7738		2.4	unknown
		*CG9975*	CG9975	2R	2.3	unknown
		*CG17180*	CG17180	3L	2.3	vesicle-mediate transport
		*CG12009*	CG12009	3L	2.2	chitin binding
		*CG8931*	CG8931	X	2.2	binding; transport
		*CG17230*	CG17230	3R	2.2	unknown
		*CG31163*	CG31163	3R	2.1	SH3/SH2 adaptor activity
		*epsilonCOP*	CG9543	2L	2.1	retrograde vesicle-mediated transport
		*CG30392*	CG30392	2R	2.1	glycolipid transporter activity
		*CG31639*	CG31639	2L	2.1	
		*CG5343*	CG5343	2L	2.0	muscle development; dendrite morphogenesis
		*CG4845*	CG4845	3R	2.0	phagocytosis; immune response
		*Hs protein cognate 2*	CG7756	3R	2.0	unfolded protein binding; ATP binding
						
	Female	*CG1368*	CG1368	X	3.9	structural constituent of chorion
		*Larval serum protein 1 α*	CG2559	X	3.0	nutrient reservoir activity; oxygen transporter
		*Arginine kinase*	CG32031	3L	3.0	arginine kinase activity
		*CG15369*	CG15369	X	2.4	cysteine protease inhibitor activity
		*CG32695*	CG32695	X	2.4	unknown
		*CG7816*	CG7816	3R	2.3	metal ion transmembrane transporter
		*white*	CG2759	X	2.2	transmembrane receptor activity; eye pigment
		*CG32850*	CG32850	4	2.1	protein binding; zinc ion binding
		*CG14717*	CG14717	3R	2.0	hydrolase activity
		*CG17118*	CG17118	2L	2.0	dendrite morphogenesis

Significant somatic, sex-differential expression determined using F-tests, and a *t*-test of means comparing expression data from male and female *tud *progeny. All genes listed had at least a 2-fold difference in transcript abundance between the sexes. For the full lists of sex-differentially expressed genes, see Additional file 3. * Annotation symbol from Flybase. ^† ^Chromosomal arm on which the gene is located. ^‡ ^Fold change difference between the sexes. ^ξ^GO annotations for each gene as listed in Flybase.

At the statistical threshold used for the *t*-tests (*q *< 0.15), 242 of the 258 genes identified by F-tests showed significant, somatic, sex differential-expression at a minimum of one time point. Close to half of these genes (119 genes) displayed somatic, sex-differential expression at only one or two time points, suggesting that they are likely to mediate discrete, sex-specific, developmental events. *RNA on the X 1 *(*roX1*) is male-biased and one of only thirteen genes that were sex-differentially expressed at all five time points examined. *roX1 *and a gene *RNA on the X 2 *(*roX2*) produce non-coding RNAs that are components of the dosage compensation macromolecular structure [17]; *roX2 *was either expressed exclusively in males or four times higher in males than females at each of the five time points examined. Dosage compensation is the process in which genes on the single X chromosome in males undergo increased transcription, which results in roughly equal amounts of mRNA product produced by the two X chromosomes in females [reviewed in [18]]. Of the additional 12 genes (three and nine with male- and female-biased expression, respectively) with sex-differential expression at all five time points examined (*Microsomal glutathione S-transferase-like*, *Glutathione Synthetase*, *CG4586*, *Larval serum protein 1 alpha*, *CG15369*, *CG15347*, *CG31775*, *Ilp6, CG1702*, *Succinate dehydrogenase B, CG7430*, and *cabut*), eight are located on the X chromosome. *Sex-lethal *(*Sxl*), the gene at the top of the sex-determination hierarchy (see Figure 1, [reviewed in [3]]), displays female-biased expression at four of the five time points examined. Interestingly, *CG3056*, a gene with male-biased expression at four of the five time points, is a paralog of *Sxl *[19]; the product of this gene may underlie additional sex-differential splicing that regulates sex-specific development.

The differences in transcript abundances observed at each stage are due to biological differences and not poor quality data, as the microarray data showed high correlation among experimental replicates and similar numbers of genes had expression data at each time point in both sexes (see Additional file 4 for all microarray correlations and number of genes expressed in each experiment).

### Time course experiment: cluster analyses

**Figure 3:**
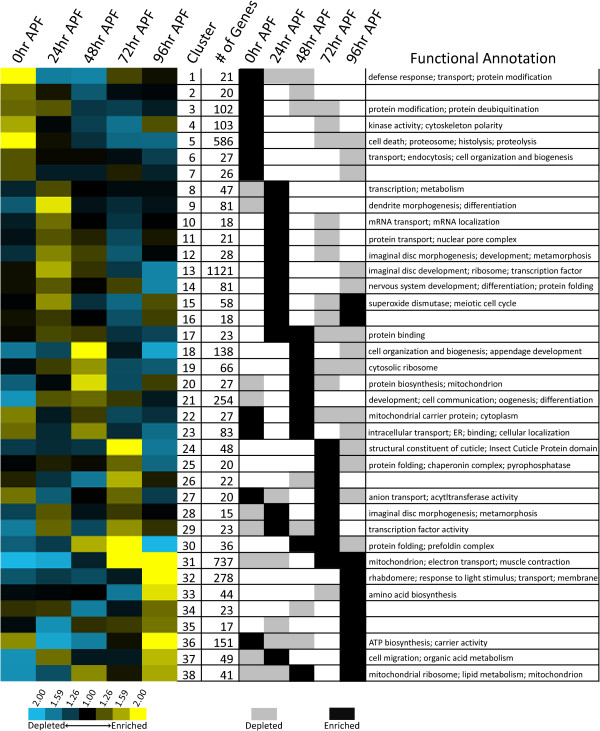
**Clusters of genes with similar expression profiles in somatic tissues during metamorphosis**. Clusters were generated using gene expression data from male and female *tud *progeny at five time points during metamorphosis, indicated at top of clustergram. Expression profiles for each cluster were generated by averaging the gene expression data at each time point for every gene in the cluster in both sexes. Yellow and blue indicates high and low levels of expression compared to a common reference, respectively. To the right of the clustergram, black and grey indicates a cluster in which gene expression is at a peak (enriched) or at a trough (depleted), respectively, relative to the average expression value across all time points. Clusters are at a peak or trough of expression if average expression was 2/3 of a standard deviation above or below the mean expression value of the five time points, respectively. Functional annotation represents functional categories that were overrepresented among the genes in the cluster, as determined by the program DAVID (*P *< 0.05 [22]).

One of the primary goals of this study was to identify genes that direct the patterning and morphogenesis of sexually dimorphic, somatic tissues. Hierarchical clustering, an algorithm that groups genes based on the similarity of their expression profiles [20], was performed using the five time points of *tud *progeny expression data to identify genes with similar expression profiles in somatic tissues. Because the numbers of expected clusters were unknown, other clustering methods, including K-means clustering and self-organizing maps [reviewed in [21]], were not employed. Thirty-eight clusters, each with a greater than 0.80 average Pearson's correlation in gene expression profiles and containing 15 genes or more, were identified and further analyzed (Figure 3 and Additional file 5). Combined, these clusters contained 4410 genes, including 82 genes with alternative transcripts expressed in two clusters and four genes with alternative transcripts expressed in three clusters. There is a high degree of separation among the clusters, with an average correlation of 0.026 between all of the clusters, demonstrating that the expression profiles for genes within a cluster are not similar to expression profiles for genes in other clusters and thus each should be considered separately in this study (see Methods).

An analysis of the expression data across the five time points for the genes in each of the 38 clusters identified 51 peaks and 49 troughs of gene expression within the clusters (Figure 3; Methods). Based on a re-sampling analysis, each of these 51 peaks and 49 troughs was significantly different than the average expression at all time points in the cluster from which they were identified (*P *< 0.01; see Methods). When wild type expression data was incorporated into the cluster analyses, gene expression profiles appear similar in wild type and *tud *progeny experiments, based on visual inspection (Additional file 6); only genes with expression in *tud *progeny were included in the cluster analyses. Furthermore, a statistical comparison of the average *tud *progeny expression data to the average wild type expression data within each cluster showed that 31 of the 38 clusters had correlations >0.70, demonstrating a high degree of similarity of expression for genes in *tud *progeny and wild type animals. The seven clusters with correlations <0.70 between the average *tud *progeny and wild type expression data contained only 172 of the 4410 genes present in the 38 clusters. Therefore, for most genes examined, the pattern of gene expression in somatic tissues during metamorphosis does not appear to be largely influenced by the presence of the germline.

To functionally analyze these clusters, the sets of genes from each cluster were examined using the program DAVID, which identifies overrepresented functional groups among the genes in each cluster, as compared to all the genes represented on the array platform (see Methods; Additional file 7; DAVID is the Database for Annotation, Visualization and Integrated Discovery [22]). To confirm the DAVID results, an independent tool that searches for enrichment of Gene Ontology terms (FlyMine; [23]) was used to assess overrepresented functional categories and gave very similar results (see Methods for details).

### Gene expression during metamorphosis

At the white pre-pupal stage (wpp; 0 hour APF), the fly is transitioning from a wandering larva into an immobile pre-pupa. The pre-pupal animal initiates the major larval-to-adult transition in several discrete ways: 1) strictly larval tissues are destroyed and replaced by corresponding adult tissues [reviewed in [24]], 2) imaginal discs and rings begin to give rise to adult structures including eyes, antennae, wings, legs, and genitalia [reviewed in [25]], 3) histoblast nests proliferate in number and give rise to non-imaginal disc derived adult epidermal structures [26], and 4) the larval central nervous system is remodeled through the destruction of some larval neurons, proliferation of neuroblasts to generate new neurons, and remodeling of some larval neuronal projections [reviewed in [27]]. The 24 hour APF time point is between the pre-pupal pulse of ecdysone, which peaks at 12 hour APF and triggers head eversion, and the large pupal pulse of ecdysone that initiates around 24 hour APF [reviewed in [1]]. By 24 hour APF the majority of larval-specific tissues are degraded and adult development is triggered [reviewed in [28]]. During the time between the 24 to 48 hour APF stages, the imaginal discs are still undergoing morphogenesis, but are close to their final adult form. The wings, leg muscles, abdominal bristles, abdominal muscles and internal genital ducts are all well formed, while further development of the eyes, legs, wings, thorax, and abdomen is occurring [reviewed in [2]]. During the later stages of metamorphosis (72 hour APF), many of the tissues and structures developing in the pupae are close to their final adult form [reviewed in [27,29]]. By 96 hour APF, the pupa is within a few hours of eclosion, or emergence of the adult fly [reviewed in [30]].

To understand the transcriptional basis of these complex developmental events, expression data was analyzed in the following ways: first, genes with similar expression patterns in both male and female somatic tissues were identified based on the hierarchical cluster analyses (Figure 3). Second, clusters were identified that contained genes that either had a peak or trough of their transcript abundance at each time point (see Methods). At the 0 hour APF stage, Cluster 5 (586 genes) has genes with peak expression and was enriched for genes that encode proteins that function in the proteosome (34 genes; *P *= 6.8 × 10^-31^), have cell death activities (35 genes; *P *= 4.2 × 10^-7^) or peptidase activities (90 genes; *P *= 5.4 × 10^-18^) and thus likely function in the histolysis of larval tissues. Cluster 21 (254 genes) has genes in a trough of expression and is enriched for genes whose products function in development (60 genes; *P *= 4.2 × 10^-6^), differentiation (25 genes; *P *= 4.6 × 10^-4^), and cell communication (54 genes; *P *= 9.8 × 10^-5^), suggesting that a large fraction of genes that function in these patterning and developmental processes are at low transcript levels immediately after pre-pupal formation.

At the 24 hour APF stage, the largest cluster identified, Cluster 13 (1,121 genes), shows peak transcript abundance (Figure 3). Cluster 13 is overrepresented with genes encoding products that are annotated as functioning in imaginal disc morphogenesis (73 genes; *P *= 2.1 × 10^-15^), neurogenesis (58 genes; *P *= 3.9 × 10^-14^), programmed cell death (62 genes; *P *= 9.9 × 10^-11^), nervous system development (122 genes; *P *= 2.4 × 10^-18^), and transcription (183 genes; *P *= 2.8 × 10^-16^), demonstrating that at about 24 hour APF, many genes that drive morphogenesis and patterning have reached a peak in their transcript abundance, marking this period as critical for patterning and morphogenesis.

At the 48 hour APF stage, Cluster 18 and Cluster 21 contain 138 and 254 genes, respectively, and show peak levels only at this stage (Figure 3). Cluster 18 is enriched with genes whose products function in cell organization and biogenesis (21 genes; *P *= 0.016), appendage morphogenesis (5 genes; *P *= 0.032), and pupal development (8 genes; *P *= 0.041), suggesting that although the rudimentary adult structures are formed, there are still many structural changes taking place. Consistent with this idea, Cluster 21 is enriched with genes whose protein products function in development (60 genes; *P *= 4.2 × 10^-6^), cell communication (54 genes; *P *= 9.8 × 10^-5^) and morphogenesis (30 genes; *P *= 1.1 × 10^-4^).

At the 72 hour APF stage, genes in Cluster 31 (737 genes, Figure 3), which are in a trough at the 0 and 24 hour APF stages, quickly increase in transcript levels to ultimately peak at 72 hour APF. Cluster 31 is overrepresented with genes that encode products that function in the mitochondria (153 genes; *P *= 1.4 × 10^-77^).

One large cluster, Cluster 32 (278 genes; Figure 3) contains genes that showed a sharp rise in transcript levels at 96 hour APF, but no peaks early in metamorphosis. This cluster is enriched for genes encoding products that function in the response to light stimulus (7 genes; *P *= 6.6 × 10^-4^), visual perception (7 genes; *P *= 0.0050), and rhabdomere function (6 genes; *P *= 3.0 × 10^-5^), all of which are critical for proper vision and development of the adult eye. On the other hand, the largest cluster, which is enriched for genes that encode products functioning in developmental processes (all *P *= 3.9 × 10^-14^) and peaked in transcript abundance at 24 hour APF, is in a trough of transcript levels at 96 hour APF (Cluster 13, 1,193 genes), consistent with the idea that morphogenesis is largely complete by the pharate adult stage.

### Sex-biased gene expression in germline tissues during metamorphosis

Genes with expression during metamorphosis in either male or female germline tissues were identified using two independent F-statistic analyses (*q *< 0.15 for each test; see Methods; see Additional file 2 for numbers of genes with significant expression differences). This statistical approach also identifies genes whose expression in somatic tissues is dependent on the presence of male or female germline tissues, respectively; these two classes of genes cannot be distinguished in this study. The gene sets that are expressed within or dependent on the presence of the germline in males and females are referred to as the pupal male- and female-biased germline sets, respectively. Sets of 659 and 342 genes with male- and female-biased expression in the germline, respectively, were identified (Additional files 8 and 9). Both the male- and female-biased pupal germline sets had significant overlap with genes previously identified as highly expressed in adult male and female germline tissues, respectively (*P *< 1.1 × 10^-4^, hypergeometric test for both sets; [14]). Five-hundred-forty-three genes identified in the pupal male germline set were on the previous study's array platform [14]. Of those 543 genes, 392 were highly expressed in the adult male germline. Similarly, 305 genes identified here as being expressed in the pupal female germline were present on the previous study's array platform. Forty-seven of those 305 genes were also highly expressed in the adult female germline.

**Figure 4:**
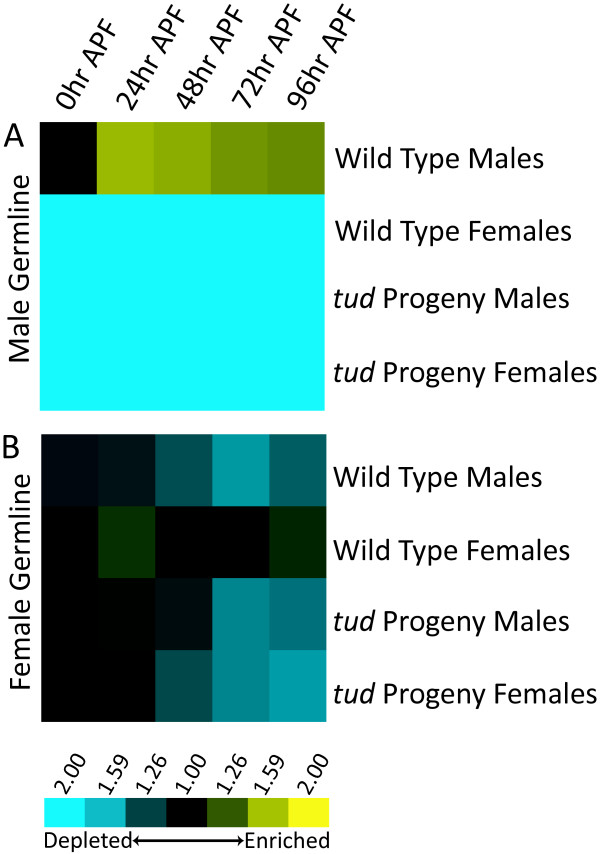
**Expression profiles of genes expressed in the male and female germline during metamorphosis**. Expression profiles were generated by averaging the gene expression data of all genes that have high expression in the male or female germline at each time point. The data for these genes for following genotypes were averaged separately: male *tud *progeny, female *tud *progeny, wild type (CS) males, and wild type (CS) females. Yellow and blue indicates high and low levels of expression compared to a common reference, respectively.

Gene expression in the male germline has already initiated at the start of metamorphosis, and by 24 hour APF has reached its peak level of gene expression; this high level of expression lasts throughout metamorphosis (Figure 4A). Sixty-nine of the 659 genes in the pupal male germline set encode products that function in the mitochondria (*P *= 5.8 × 10^-19^; DAVID analysis), consistent with the essential role for mitochondria in spermatid development and adult function [reviewed in [31]]. One-hundred-fifty-one genes (of 543 genes, 28%) were identified that are expressed in the male germline during metamorphosis, but were not previously identified as expressed in the adult male-germline (present on platform of previous study, but not significantly differentially expressed [14]), suggesting that there is pupal-specific, male-germline gene expression that might underlie male germline development.

Previously, it was observed that most genes expressed in the female germline showed the first post-embryonic peak of transcript abundance during adult stages [7]. However, because our previous study did not have data from pupal stages examining gene expression in each of the sexes separately, or in male and female *tud *progeny, we were unable to definitively identify the genes expressed in the female germline at pupal stages. The data presented here demonstrate that are a substantial number of genes with female-biased germline expression during pupal stages (Figure 4B). Between the 48 and 72 hour APF stages, the structures derived from the female genital disc establish connections with female gonadal tissues to form the female reproductive system [reviewed in [32]]. The development of female reproductive structures likely requires gene expression in both somatic and germline tissues. This idea is consistent with the functions of genes with pupal female germline expression, as this set is overrepresented with genes that function in developmental processes (76 genes, *P *= 0.0031; DAVID analysis).

Interestingly, the transcript level of genes expressed in the pupal female-germline also peaks in both wild type males and females and *tud *progeny males and females at the early stages of metamorphosis (Figure 4B), suggesting they also play a non-sex-specific role in pupal somatic tissues. Several genes encoding products annotated as functioning in the female germline (found in Cluster 21) peak in transcript abundance in male and female somatic tissues at 48 hour APF (Figure 3). However, by the later stages of metamorphosis, the levels of these transcripts remain high only in wild type females and drop to trough levels in wild type males, and *tud *male and female progeny.

The chromosomal distribution of genes with sex-biased expression in the male and female germlines was additionally analyzed. Genes expressed in the pupal male germline are underrepresented on the X chromosome and overrepresented on the left arm of the second chromosome (*P *= 3.5 × 10^-7 ^and 0.0072, respectively, hypergeometric test), both of which have been shown for genes expressed in adult male germline tissues [14]. Interestingly, genes expressed in the pupal male germline are also overrepresented on the right arm of the third chromosome (*P *= 0.024, hypergeometric test).

### Global transcriptional profiles during metamorphosis

**Figure 5:**
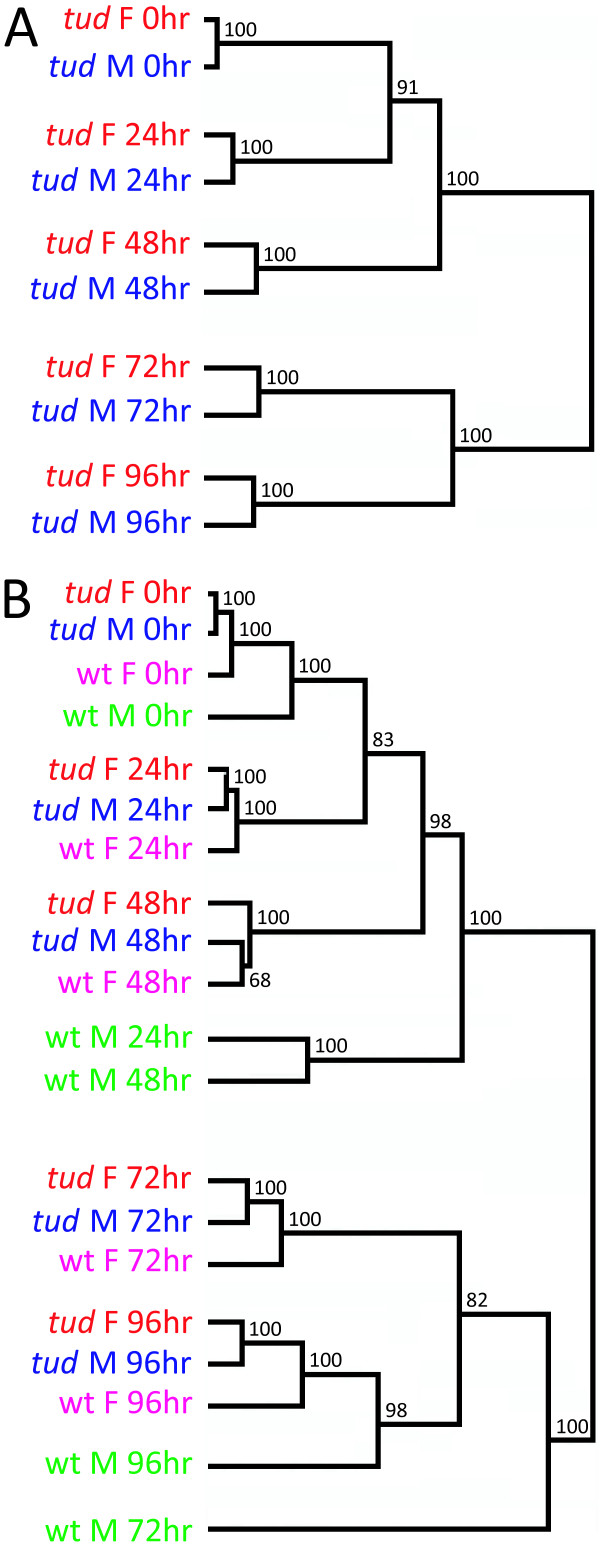
**Cluster of global expression profiles for Drosophila transcripts across metamorphosis**. Dendrogram shows the similarity across transcriptional profiles at five time points during metamorphosis (0, 24, 48, 72, and 96 hour APF) for (A) male *tud *progeny (blue) and female *tud *progeny (red) and (B) male *tud *progeny, female *tud *progeny, wild type (CS) males (green), and wild type (CS) females (violet). Hierarchical clustering and Pearson correlation distance measure was used to group experiments based on their global expression profile using all expression data for annotated genes from each array. Values on each node represent the confidence of the separation (approximately unbiased *P *value) derived using multiscale bootstrap resampling and the program Pvclust [63].

Hierarchical clustering was performed using all the data from each microarray experiment from the time course study, rather than using the data from each gene, to determine how similar global expression patterns are between males and females. When the *tud *progeny expression data was analyzed, the global expression profiles of males and females from each pupal time point were most similar to each other (Figure 5A). This was expected because very few genes with somatic, sex-differential expression were identified (see above). A clear distinction between overall gene expression at early stages (0–48 hour APF pupae) and late stages (72–96 hour APF pupae) was observed (Figure 5A). This is consistent with our cluster analyses (Figure 3), where many genes appear co-regulated at either early or late stages of metamorphosis, but not at both early and late stages: 1,745 genes shared either peaks or troughs of transcript levels at multiple early stages (0–48 hour APF) or late stages (72–96 hour APF), while only 513 genes shared peaks or troughs of transcript levels at an both an early and a late time point.

When the wild type expression data and the *tud *progeny expression data were analyzed together, the clear distinction between early and late metamorphosis remained, as with the *tud *progeny expression data alone (Figure 5B). As expected, male-germline gene expression has a large effect on how the global transcriptional profiles cluster, with wild type males expression data always clustering separately from wild type females and from the male and female *tud *progeny. This effect appears to be less substantial at 0 hour APF, as the global expression profile of wild type males clusters closest to the data from the other three genotypes, suggesting that at the start of metamorphosis, many genes expressed in the male germline are not as abundant during early time points. The wild type female array experiments also cluster closely to, but separately from, the *tud *progeny array data, with the largest differences seen at 72 and 96 hour APF. This is consistent with gene expression in the female germline increasing at the end of metamorphosis (Figure 4B).

### Sex hierarchy-regulated somatic sex-differential expression

Next, genes regulated by the sex hierarchy during pupal developmental stages were identified. Nearly all of the sexually dimorphic tissues are either patterned or undergoing morphogenesis to bring about the adult sexual dimorphisms during pupal stages. The 48 hour APF pupal stage was chosen because previous studies showed that FRU^M ^peaks at this stage [33] and DSX shows high expression at this time [34]. For these experiments, the array hybridizations were performed as direct comparisons using RNA from the two genotypes (see Table 1 and Methods). Genes were first identified that had sex-biased transcript levels between wild type males and females by analyzing expression data from two different wild type strains, Canton S and Berlin (*q *< 0.15), and from *tud *progeny males and females (one-tailed *t*-test, *q *< 0.15). This resulted in a set of 420 genes (320 and 100 genes with female- and male-biased expression, respectively; Additional file 10). This is substantially more than was identified in the time course analysis for this time point (79 genes, see above); however this difference is likely due to the increased number of replicates (four replicates for each comparison versus three replicates in time course) and the decreased statistical error by directly comparing gene expression on the same array, as opposed to using a common reference RNA sample.

Given the larger number of somatic, sex-differentially expressed genes identified by this approach, it could be determined if there was a bias for chromosomal positions in these gene sets. Genes with somatic male-biased transcript levels at the adult stage are known to be underrepresented on the X-chromosome [14]. There was not a similar bias for the 100 genes with somatic, male-biased transcript levels at the 48 hour APF pupal stage, but rather there was an equal distribution across all chromosomes (hypergeometric test, *P *> 0.05). Interestingly, the genes with somatic female-biased transcript levels at this pupal stage were overrepresented with genes located on the X chromosome (90 genes, hypergeometric test, *P *< 0.001). The previous study did not find any significant over- or underrepresentation on any chromosome for genes with adult somatic female-biased expression in the adult [14].

### Genes differentially expressed as a consequence *tra*

Next, genes were identified that were differentially expressed as a consequence of *tra*, a gene in the sex hierarchy that encodes a pre-mRNA splicing factor required for the production of the sex-specific *dsx *mRNA splice variants [35]. Transcript levels in chromosomally XX flies mutant for *tra *(hereafter called *tra *pseudomales) were compared to wild type female flies. The *tra *pseudomales produce DSX^M ^and look very similar to wild type males. Of the 420 genes that showed somatic sex-biased transcript levels, 95 genes were identified (72 female-biased and 23 male-biased) that are also significantly different between *tra *pseudomales and wild type females (one-tailed *t*-test, *q *< 0.15 for each test, Additional file 10). In this experimental design genes were required to be differentially expressed in three different genotype comparisons of male and female gene expression (CS, Berlin, and *tud*), precluding the identification of genes that are differentially expressed in only one strain. This is also true for the set of DSX-regulated genes identified below. As a validation of our experimental approach, *Sxl*, *tra*, *roX1*, and *roX2 *are all sex-differentially expressed in somatic tissues (wild type and *tud *progeny comparisons). Only *tra *is differentially expressed in the chromosomally XX, *tra *pseudomale and wild type female comparison. This is expected as *Sxl*, *roX1*, and *roX2 *are not regulated downstream of *tra *in the sex determination hierarchy, but are regulated downstream of the primary determinate of sex, the X chromosome to autosome ratio (Figure 1; [reviewed in [36]]).

Of the 326 genes that are not regulated by TRA, a large portion (169) may be false negatives as their expression values are significant or close to significantly different (*q *< 0.30) in microarray experiments identifying genes regulated by *dsx *(see below). A gene regulated downstream of *dsx *should also be regulated downstream of *tra*. Another 19 have a *q *value close to the cutoff for significance in the *tra *microarray expression data (*q *< 0.30). Removing these 188 genes from consideration still leaves a large number of genes (138 genes) that are sex-differentially expressed independently of *tra*. A significant number of these 138 genes (45 genes; *P *< 0.05, hypergeometric test) are located on the X chromosome. It is possible that differences in transcript levels of these genes is due to differences downstream of *Sxl*, or X chromosome composition in males and females, suggesting that the dosage compensation process does not completely normalize expression between males and females for all genes on the X chromosome.

### Genes differentially expressed as a consequence *dsx*

Next, gene expression between pupae that are transheterozygous for the *dsx*^*D *^allele [11] – an allele that only produces the male-specific isoform (DSX^M^) – and a *dsx *null deletion allele (*dsx*^*m*+*r*15^) was compared to gene expression in wild type females. These chromosomally XX, *dsx*^*D*^/*dsx*^*m*+*r*15 ^pseudomales look very similar to wild type males, as they only produce DSX^M^. Of the 95 genes that are sex-differentially expressed in somatic tissues and downstream of *tra*, 66 genes were identified as being regulated downstream of *dsx *(one-tailed *t*-test, *q *< .015). Forty-six and 20 genes are more highly expressed in females and males, respectively, downstream of *tra *and *dsx *(Table 3 and Additional file 10).

**Table 3:** Genes expressed downstream of DSX at 48 hour APF.

**Sex-bias**	**Gene Name**	CG # *	**Ch**^†^	**FC**^‡^	**Functional Annotation**^ξ^
Male	*CG1342*	CG1342	3R	6.0	serine-type endopeptidase inhibitor activity
	*Osiris 11*	CG15596	3R	4.9	unknown
	*CG12063*	CG12063	3R	4.8	unknown
	*CG13535*	CG13535		4.2	unknown
	*SP71*	CG17131	X	4.0	unknown
	*CG32159*	CG32159	3L	6.5	unknown
	*Ecdysone-inducible gene E1*	CG32356	3L	4.8	lipoprotein binding; imaginal disc eversion
	*CG4386*	CG4386	2R	4.5	trypsin activity; proteolysis
	*miniature*	CG9369	X	3.8	cuticle pattern formation; epidermal cell differentiation
	*Osiris 21*	CG14925	2L	3.4	unknown
	*CG4702*	CG4702	3R	3.3	unknown
	*CG8420*	CG8420	3R	3.2	unknown
	*dusky-like*	CG15013	3L	3.1	structural constituent of cuticle
	*CG1499*	CG1499	3R	3.1	unknown
	*Ecdysone-inducible gene L1*	CG10717	3L	3.1	unknown
	*ectodermal*	CG6611	3L	3.0	unknown
	*vrille*	CG14029	2L	3.0	transcription factor activity; bristle morphogenesis
	*CG13728*	CG13728	3L	2.9	unknown
	*CG15020*	CG15020	3L	2.8	structural constituent of cuticle
	*CG15643*	CG15643	X	2.8	unknown
	*CG32354*	CG32354	3L	2.7	endopeptidase inhibitor activity
	*CG13078*	CG13078	2L	2.7	unknown
	*CG15589*	CG15589	3R	2.7	unknown
	*shavenoid*	CG13209	2R	2.7	wing hair biogenesis; antennal morphogenesis
	*CG10898*	CG10898	3R	2.5	DNA repair
	*CG13059*	CG13059	3L	2.3	unknown
	*CG4844*	CG4844	2R	2.3	unknown
	*CG31637*	CG31637	2L	2.2	sulfotransferase activity; carbohydrate metabolism
	*CG11438*	CG11438	3L	2.1	phosphatidate phosphatase activity; lipid metabolism
	*Dopa decarboxylase*	CG10697	2L	2.0	catecholamine metabolism; learning and/or memory
	*brother of iHog*	CG32796	X	2.0	ectoderm development; nervous system development
	*CG10249*	CG10249	2R	2.0	unknown
					
Female	*CG6337*	CG6337	2R	15.2	cysteine-type endopeptidase activity
	*CG31878*	CG31878	2L	11.9	structural constituent of cuticle
	*CG16884*	CG16884	2L	11.0	unknown
	*CG16885*	CG16885	2L	10.2	unknown
	*CG14752*	CG14752	2R	9.0	unknown
	*CG8927*	CG8927	3R	7.5	unknown
	*CG7330*	CG7330	3L	6.7	unknown
	*CG15251*	CG15251	X	6.5	unknown
	*CG12164*	CG12164	2R	6.1	unknown
	*CG10264*	CG10264	3R	6.0	unknown
	*Cuticular protein 97Eb*	CG15884	3R	5.9	structural constituent of cuticle
	*CG32694*	CG32694	X	5.6	unknown
	*CG16886*	CG16886	2L	5.3	unknown
	*b6*	CG3100	X	5.2	neuronal pentraxin receptor activity
	*Peritrophin A*	CG17058	X	5.2	chitin binding; chitin metabolism
	*CG10175*	CG10175	3R	4.9	carboxylesterase activity; phosphopantetheine binding
	*CG30427*	CG30427	2R	4.9	oxidoreductase activity
	*CG11380*	CG11380	X	4.8	unknown
	*CG15778*	CG15778	X	4.7	unknown
	*CG17829*	CG17829	X	4.5	nucleic acid binding; zinc ion binding
	*CG1441*	CG1441	2R	4.3	oxidoreductase activity
	*CG32645*	CG32645	X	4.3	transferase activity
	*CG32036*	CG32036	3L	4.2	chitin binding; chitin metabolism
	*CG14052*	CG14052	X	4.1	unknown
	*Cyp303a1*	CG4163	2L	4.1	sensory organ development; steroid metabolism
	*CG3244*	CG3244	2L	4.0	sugar binding
	*CG32499*	CG32499	X	3.8	chitin binding; chitin metabolism
	*CG32550*	CG32550	X	3.7	unknown
	*CG10051*	CG10051	2R	3.7	unknown
	*CG13931*	CG13931	3L	3.6	unknown
	*Pherokine 3*	CG9358	2R	3.6	protein kinase activity; gametogenesis
	*CG17707*	CG17707	X	3.5	unknown
	*CG14534*	CG14534	2L	3.5	nutrient reservoir activity
	*Cuticular protein 50Ca*	CG13338	2R	3.5	structural constituent of cuticle
	*CG13138*	CG13138	2L	3.5	unknown
	*CG9021*	CG9021	2L	3.4	unknown
	*GV1*	CG12023	3L	3.4	DNA binding
	*CG14770*	CG14770	X	3.4	unknown
	*CG15322*	CG15322	X	3.3	unknown
	*CG7031*	CG7031	3R	3.3	unknown
	*Cuticular protein 97Ea*	CG6131	3R	3.3	structural constituent of cuticle
	*CG14218*	CG14218	X	3.3	serine-type endopeptidase activity
	*CG33299*	CG33299	2L	3.3	unknown
	*CG17777*	CG17777	X	3.2	unknown
	*CG15055*	CG15055	X	3.2	unknown
	*CG1561*	CG1561	X	3.1	unknown
	*CG1702*	CG1702	X	3.0	glutathione transferase activity; defense response
	*CG12057*	CG12057	X	2.9	unknown
	*CG13616*	CG13616	3R	2.9	unknown
	*CG17032*	CG17032	3L	2.9	unknown
	*CG6592*	CG6592	3L	2.9	chymotrypsin activity; proteolysis
	*HDC15381*	CG33342	3R	2.8	unknown
	*Cuticular protein 51A*	CG10112	2R	2.8	structural constituent of cuticle
	*CG8192*	CG8192	2R	2.8	unknown
	*CG5873*	CG5873	3R	2.7	peroxidase activity; defense response
	*Peroxidase*	CG3477	3R	2.7	peroxidase activity; phagocytosis, engulfment
	*CG14946*	CG14946	2L	2.6	oxidoreductase activity; binding
	*LCBP1*	CG8756	3L	2.6	chitin deacetylase activity; open tracheal system
	*SRY interacting protein 1*	CG10939	2R	2.5	cell homeostasis; ion transport; olfactory behavior
	*flightin*	CG7445	3L	2.5	muscle thick filament assembly
	*obstructor-A*	CG17052	X	2.5	chitin binding; chitin metabolism
	*Gfat1*	CG12449	3R	2.5	sugar binding; carbohydrate biosynthesis
	*CG6739*	CG6739	2L	2.5	unknown
	*CG13062*	CG13062	3L	2.5	unknown
	*CG5506*	CG5506	3L	2.4	unknown
	*dpr13*	CG33996	2R	2.4	unknown
	*CG7047*	CG7047	3L	2.4	unknown
	*CG9850*	CG9850	2R	2.4	metallopeptidase activity; cell proliferation
	*CG30101*	CG30101	2R	2.4	unknown
	*omega*	CG32145	3L	2.4	peptidase activity; signal transduction
	*CG4404*	CG4404	X	2.4	DNA binding
	*lethal (2) essential for life*	CG4533	2R	2.3	embryonic development; protein folding
	*CG32816*	CG32816	X	2.3	unknown
	*CG14191*	CG14191	X	2.3	unknown
	*CG7135*	CG7135	X	2.2	unknown
	*pgant2*	CG3254	2L	2.2	protein amino acid glycosylation
	*CG8358*	CG8358	3R	2.1	neprilysin activity; proteolysis; signal transduction
	*CG12523*	CG12523	3L	2.1	unknown
	*melted*	CG8624	3L	2.1	specific transcriptional repressor activity; development
	*CG9134*	CG9134	3L	2.1	sugar binding
	*CG9328*	CG9328	2L	2.1	unknown
	*serpentine*	CG32209	3L	2.1	chitin metabolism; tracheal system development
	*CG4484*	CG4484	3L	2.1	sucrose:hydrogen symporter activity
	*CG16820*	CG16820	2L	2.1	unknown
	*CG9990*	CG9990	3R	2.1	ATPase activity; transporter activity; ATP binding
	*CG13188*	CG13188	2R	2.1	unknown
	*Limpet*	CG32171	3L	2.0	transcription factor activity; development

All genes listed had at least a 2.0-fold difference in transcript abundance when comparing XX *dsx*^*D *^pseudomales to wild type females at 48 hour APF. For the full lists of genes regulated as a consequence of *dsx*, see Additional file 10. * Annotation symbol from Flybase. ^† ^Chromosomal arm on which the gene is located. ^‡ ^Fold change difference between the sexes. ^ξ^GO annotations for each gene as listed in Flybase.

Aside from the *tra *gene itself, 28 genes were differentially regulated by TRA, but were not differentially expressed between *dsx*^*D *^and wild type females. If genes that are close to the significance level (*q *< 0.30; 6 genes) and genes with expression data in only one or two *dsx*^*D *^comparisons (3 genes) are removed, 19 genes remain that are downstream of *tra*, but not *dsx*. Interestingly, none of these genes are significantly differentially expressed in similar experiments examining FRU^M ^regulation at this stage (data not shown). This suggests either an alternate branch of the sex-hierarchy downstream of *tra*, possibly through *dissatisfaction *[37], or the possibility of additional genes on which TRA acts to sex-specifically splice their pre-mRNAs, leading to differential abundance of transcripts due to differences in mRNA stability.

Requiring a gene to show statistical differences in expression in all direct microarray experiments yields a high confidence set of true positives regulated downstream of *dsx*, but will likely generate false negatives. To identify additional *dsx *regulated genes that might have been missed because of the stringency of having to pass multiple tests, genes were included that showed sex-differential, somatic expression, but which were not differentially expressed in the *tra *microarray comparisons. These genes were required to be significantly differentially expressed in the *dsx*^*D *^comparisons at a more stringent level (*q *< 0.05) to avoid false positives. This yielded an additional 107 genes, with 75 and 32 showing female- and male-biased expression, respectively (Table 3 and Additional file 10). This study thus identified 173 genes regulated as a consequence of *dsx *(DSX set; 121 and 52 genes with female- and male-biased expression, respectively).

Several of the genes with male- and female-biased expression in the DSX set with the highest fold change include those with products that might be involved in epithelial morphogenesis, imaginal disc morphogenesis or cuticle formation, based on their sequence identity. The 52 genes with male-biased expression contains seven such genes, including ecdysone inducible *ImpE1 *(FC = 4.8), *miniature *(FC = 3.8), and *dusky-like *(FC = 3.1). Among the 15 genes with the highest female-biased expression, four encode proteins with cuticular domains (*Cuticular protein 97Eb*, *50Ca*, *97Ea*, and *51A*; FC = 5.9, 3.5, 3.3, and 2.8, respectively), as well as *obstructor-A *(FC = 2.5) and *abdominal A *(FC = 1.6). While it has long been recognized that cuticle deposition is tied to tissue morphogenesis and both are developmental events occurring during the middle of metamorphosis, the identification of several genes likely involved in sex-specific aspects of this process had not been determined until this study.

Six genes with female-biased expression regulated downstream of DSX at 48 hour APF have products with functions in the muscle or muscle differentiation (*flightin, Limpet*, *CG31781*, *Tropomyosin 1*, *abdominal-A *and *Sarcoplasmic calcium-binding protein *[7,38-42]). This suggests that aspects of pupal muscle development occur in temporally distinct manner between males and females, and that this differential timing is regulated by DSX. It is not clear is if this is due to the development of sex-specific muscles or due to differences in the developmental rate of non-sex-specific muscles between males and females.

### Characterization of the modes of DSX regulation

In our previous microarray study examining modes of DSX-regulated gene expression at the adult stage in head tissues, a large number of sex-biased genes were found that were either activated or repressed as consequence of *dsx *activity in both males and females, but the extent of activation or repression was sex-specific [43,44]. This mode of regulation was distinct from the previous descriptions of DSX-regulated gene expression based on the only known direct targets of DSX, *Yolk protein 1 *(*Yp1*) and *Yolk protein 2 *(*Yp2*). DSX^F ^activates *Yp1 and Yp2 *expression in the female fat body and DSX^M ^represses *Yp1 and Yp2 *expression in the male fat body tissues [45].

To test if the set of genes regulated as a consequence of DSX activity contains genes that may be directly regulated by DSX, it was determined if the known DSX binding site sequences are present in the regulatory region of these genes [46]. We searched for the presence of two DSX binding sites in the DNA sequence 2000 base pairs upstream from the transcription start and within the first intron, for each gene in our list (see Methods for search criterion) [45]. Two DSX-binding sites were identified in 46 of the 165 (28%) DSX-regulated genes, a statistical overrepresentation as compared to all genes in the genome (*P *= 0.0002, hypergeometric test), suggesting that a fraction of the genes identified here may indeed be direct targets of DSX. We note that in this study regulation by DSX may be direct or indirect.

To determine the modes of regulation by DSX in pupal stages, gene expression was compared between chromosomally XX and XY *dsx *null flies and wild type females and males, respectively (hereafter called *dsx *null comparisons). Data was examined for the 173 genes we identified here as being downstream of *dsx *(DSX set; Table 3 and Additional file 10). Fifteen genes did not have enough expression data for statistical analysis or were not significantly differential expressed in either *dsx *null comparison and were therefore not considered for further analysis. Of the remaining 158 genes, 151 show significant differential expression (*q *< 0.15) in both *dsx *null comparisons, which suggests regulation downstream of both DSX^F ^and DSX^M ^activity. The remaining seven genes (2 and 5 male- and female-biased genes, respectively) only showed significant differential expression in one of the *dsx *null comparisons; these seven genes may possibly be regulated downstream of one isoform of DSX, a method of DSX regulation that was previously proposed for some genes with sex-differential expression in the adult [43,44].

Of the 151 genes regulated as a consequence of *dsx *in both sexes, 104 of the genes had female-biased expression and were more highly expressed in wild type females and males as compared to *dsx *null females and males, respectively. This suggests that these genes are activated downstream of DSX in both females and males, but that DSX^F ^activity results in more potent activation. Forty of the 151 genes were male-biased and more highly expressed in male and female *dsx *null flies than in wild type males and females, suggesting these genes are repressed as a consequence of DSX activity in both males and females, but DSX^F ^activity results in more potent repression. Thus, the majority of genes that are regulated as a consequence of *dsx *are not regulated in the *Yp*-like mode of regulation, but rather are regulated similarly in both sexes, with gene expression downstream of one isoform resulting in more potent activation or repression, as previously described in our studies of adult head tissues [44]. Interestingly, *Yp*-like regulation was observed for only seven genes in our pupal dataset: the genes with male-biased expression *CG8086 *and *CG14995 *and the genes with female-biased expression *abdominal-A *(*abdA*), *LpR1*, *CG10802*, *CG1441*, and *CG9485*. It is possible that the *Yp*-like mode of regulation may be the more common method of regulation for a particular class of genes (i.e., direct targets) or might be revealed to be the primary mode of *dsx *regulation when higher resolution analyses are performed.

*abdA*, which appears to be activated downstream of DSX^F ^in females and repressed downstream of DSX^M ^in males, is a well-characterized homeotic selector gene that was shown to be important for specifying segment identity [47,48]. In the time course analyses above, *abdA *was found to have female biased expression at 0, 24, 48, and 72 hour APF, with the highest expression difference between the sexes at 48 and 72 hour APF. Previous research examining 40–45 hour APF, suggested that ABD-A and DSX, along with Abdominal-B, act to regulate the expression level of a downstream target, *bric-a-brac*, and lead to differential abdominal pigmentation between males and females [49]. In that study it was shown that *abdA *transcript levels in the abdominal epidermis do not vary between *dsx *null animals and wild type animals, thus suggesting that *abdA *is not regulated by DSX in this tissue. The *dsx*-dependent differential expression of *abdA *that we observed could be due to expression in other tissues, since here whole pupae were analyzed. Indeed, *abdA *has been shown to be expressed and functional in several distinct tissues and cell types, including abdominal neuroblasts and the female genital disc [50,51].

The proposed modes of regulation were validated by additional microarray experiments in which the male and female isoforms of DSX were over-expressed (data not shown). Of the 158 genes in the DSX-regulated set for which DSX modes of regulation was examined, 47 did not show significant differential expression in the experiments when we either over-expressed DSX^F ^in females or DSX^M ^in males. Of the remaining 111 genes, 35 were male-biased; these genes showed decreased expression when DSX was over-expressed, either in one or both of the DSX isoform over-expression experiments. Similarly, of the remaining 76 female biased genes, 74 showed increased expression levels when DSX was over-expressed, either in one or both of the DSX isoform over-expression experiments. Only two genes with female-biased expression (*CG4484 *and *CG3244*) showed decreased expression levels when DSX^F ^was over expressed in females compared to control females, opposite of the predicted effect from our model.

## Conclusion

Here, an analysis of gene expression profiles underlying the developmental transitions that occur during metamorphosis in both wild type and germline-deficient males and females is presented. Many genes were identified that are expressed in somatic tissues of both males and females at five discrete times during development, with far fewer showing sex-differential expression in somatic tissues. The observation that about half of the genes identified with somatic, sex-differential expression show this pattern of expression at only one or two of the stages examined suggests that expression at these stages mediates distinct developmental events. Further molecular-genetic analyses of genes identified here will provide insight into their functional roles during development and how tissues that display little sexual dimorphism at the start of metamorphosis undergo patterning and morphogenesis to result in highly dimorphic adult structures. The analyses also identified genes expressed in either the male or female germline during pupal stages that were not previously shown to have germline-dependent expression or were not thought to be expressed as early as the pupal stages [7,15].

Differences in somatic, sex-differential expression were examined more extensively at the 48 hour APF stage. At this stage, the set of genes with somatic, female-biased transcript levels was overrepresented with genes located on the X chromosome. This could be a consequence of the increased amount of time, during the life history of *Drosophila*, that the X chromosome spends in females, resulting in a selection for genes that function in female somatic tissues on this chromosome; this idea that was previously suggested to explain the relative depletion of genes on the X chromosome that have male-biased expression [14,52].

Transcriptional differences between the sexes occurring downstream of *tra *and *dsx*, two sex determination hierarchy regulatory genes, were also examined at the 48 hour APF stage. The genes regulated as a consequence of *tra *and *dsx *that show high expression differences are overrepresented with those that encode proteins that function in epithelial morphogenesis, imaginal disc morphogenesis or cuticle formation. This set also includes the well-studied homeotic gene *abdA*. Further analyses of these genes will provide insight into how sex-specific developmental programs are integrated with other developmental programs. The observation that *abdA *is regulated as a consequence of *dsx*, suggests that during pupal stages, *dsx *might direct aspects of cell-fate identity, rather than acting in a parallel pathway to overlay sex-specific regulation information onto cells that are directed to their cell-fate identities in a non-sex-specific manner. Identifying the tissues that give rise to *abdA *sex-differential transcript abundances and determining how *dsx *regulates these differences are important future studies. The identification of genes regulated downstream of *tra *and *dsx *provides some of the first, large-scale, molecular insights into how the sex hierarchy instructs the changes that culminate in the production of sexually dimorphic adult male and females.

The data presented here broadens the idea that the *Yp*-like mode of *dsx*-regulated gene expression – in which DSX^M ^and DSX^F ^regulate a given gene with one acting as an inducer in one sex and the other acting as a repressor in the other sex – might be the case for a small set of genes [53,54]. In this study and our previous genomic studies [43,44], very few genes were identified that display that pattern. Our results extend the idea that many genes with sex-differential expression are either activated or repressed as a consequence of DSX^M ^and DSX^F ^activity in both sexes, but that the extent of activation or repression is sex-specific [44]. Therefore, for many genes, the consequence of *dsx *activity does not switch a gene on or off, but dials expression to high or low levels, resulting in sex-differential expression and ultimately sex-specific development.

This mode of gene regulation makes the most sense in cases where there are homologous structures in both males and females that undergo sex-specific modifications, like the sex-comb bristles on the foreleg in males or sex-specific differences in abdominal pigmentation, where similar sets of genes might be active in males and females, but to different extents. A similar idea was first suggested to explain foreleg bristle phenotypes in animals in which DSX was ectopically produced [55]. This mode of *dsx *gene regulation is more difficult to reconcile for cases where males and females have very different structures derived from different embryonic primordia, such as the genital disc primordia that give rise to the male and female internal and external genitalia. However, even in these tissues, similar batteries of genes might be used during metamorphosis to drive morphogenesis, but might have sex-specific spatial patterns, resulting in different transcript levels, consistent with what has been previously shown [reviewed in [56]], and consistent with our observations. It should be noted that higher resolution analyses of sex-differential gene expression for a given gene will likely reveal that the modes of *dsx*-regulated gene expression are much more complicated than can be predicted from whole animal microarray expression studies. Analyses of the spatial gene expression patterns and molecular and functional studies on the genes identified here are an important next step in understanding how *dsx*-regulated gene expression directs sex-specific development.

## Methods

### Fly collections and strains

Male and female flies were collected at the white pre-pupal stage between Zeitgeber time (ZT) 1 and ZT 4 and aged at 25°C for the following hours: 0, 24, 48, 72, and 96 (time course) or 48 (sex hierarchy mutants), and then snap frozen in liquid nitrogen. Wild type flies were Canton S (CS) and Berlin. Animals that lack germline tissues (called *tud *progeny) were the progeny of female *tud*^1^*bw*^1^*sp*^1 ^and male *y*, *w*, *P*[*w*^+*cM*^*UBI-GFP];ID-1 P [FRT(w*^*hs*^)]101. Female *tud *progeny had GFP expression and could be distinguished from males by fluorescence microscopy. Below, chromosomal sex for sex hierarchy mutant flies is indicated in parentheses. Chromosomally XX sex hierarchy mutants were identified based on the GFP marker on the X chromosome, derived paternally. *tra *pseudomales were the genotype *y*, *w*, *P *[*w*^+*cM*^*UBI-GFP]/+;tra*^1^/*Df(3L)st-j7 *(XX) and were compared to CS females. *dsx *pseudomales were *y*, *w*, *P*[*w*^+*cM*^*UBI-GFP]/+;dsx*^*D*^, *Sb*^1^, *e*^1^/*dsx*^*m*+*r*15 ^(XX), and compared to CS females. For the *dsx *null analyses, *P [w*^+*cM*^*UBI-GFP]/+; dsx*^*d*+*R*3^/*dsx*^*m*+*r*15 ^(XX) flies were compared to CS females and *dsx*^*d*+*r*3^/*dsx*^*m*+*r*15 ^(XY) flies were compared to CS males. All flies were kept at 25°C in a 12:12 hour light-dark cycle and grown using standard food media.

### Microarray experiments

All time course microarray experiments were conducted with three replicates; for every experiment, cDNA from the experimental genotype contained incorporated Cy3-labeled dUTP and cDNA from the reference sample contained incorporated Cy5-labeled dUTP. The common reference was comprised of RNA from CS flies of both sexes from all pupal stages. Microarray comparisons using RNA derived from sex determination hierarchy mutants were conducted with four replicates, with a dye-swap design; i.e. cDNA from each genotype contained incorporated Cy3-labeled dUTP in two experiments and contained incorporated Cy5-labeled dUTP in the other two experiments. RNA was isolated from ~30 pupae by homogenization and extraction using the TRIzol^® ^protocol (Invitrogen, Carlsbad, CA) and resuspended in 20 μL diethylpyrocarbonate (DEPC)-treated H_2_O.

cDNAs were directly labeled with Cy5 or Cy3 during the reverse transcription reaction; 30 μg of total RNA was used as a starting template. The reverse transcription reaction was performed for two hours at 42°C using the following reagents (values in parentheses are final molarity or final concentration): oligo dT primer (Operon, 3.75 μM), dithiothreitol (Invitrogen, 10 mM), First Strand Buffer (Invitrogen, 1×), dNTPs minus dTTP (Invitrogen, 0.5 mM), dTTP (Invitrogen, 50 μM), Cy-labeled dUTP (Perkin-Elmer, 0.625 nM), and Superscript II reverse transcriptase (Invitrogen, 10 U/μL). The reaction was stopped and RNA was hydrolyzed by a 20 minute incubation at 65°C with NaOH (167 mM) and EDTA (83 mM). After neutralizing by adding HEPES buffer (pH8.0; 294 mM) and sodium acetate (pH5.2; 228 mM), cDNA samples were purified (Gel-Purification Kit, Qiagen, Valencia, CA). cDNA samples were then dried and resuspended in formamide (56%), sodium citrate buffer (SSC, 3.37×), SDS (1.12%), Denhardts (5.62×), and Polyadenylic acid potassium salt (0.9 mg/ml; Sigma-Aldrich, St. Louis, MO). Samples were boiled for 2 minutes and then applied to microarray slides underneath a LifterSlip (Erie Scientific, Portsmouth, PA). Microarrays were hybridized at 42°C for 14–18 hours, then washed in a solution of 1.5% SDS and 1 × SSC for 5 minutes, a solution of 0.20 × SSC for 5 minutes, and two solutions of 0.05 × SSC for 10 minutes each.

### Microarray production and analysis

The oligonucleotide set that was printed on the glass slides consisted of 15,156 oligonucleotides, representing the full predicted set of transcribed regions of the *D. melanogaster *genome, including 14,454 known and predicted open reading frames and an additional 702 control spots. The 14,454 oligonucleotides represent 13,820 unique genes. Throughout the paper, the number of genes identified is reported, while the Additional files contain information on the specific transcripts for each gene, including multiple transcripts identified for the same gene. The oligomer set was designed by the International Drosophila Array Consortium (INDAC; ) based on release 4.1 of the *D. melanogaster *genome using a custom implementation of OligoArray2 [57]. The oligonucleotides were designed with sizes ranging between 65–69 nucleotides, a minimal Tm window, bias towards the 3'-ends of transcripts, and minimal sequence similarity to other genes [58]. The oligonucleotides were synthesized by Illumina (San Diego, CA); sequences can be downloaded from FlyMine: . Additionally, microarrays used for the time course analyses and the *dsx *null analyses contained additional array elements for *Sxl*, *tra*, the female-specific splice form of *dsx *(*dsx*^*F*^), the male-specific splice form of *dsx *(*dsx*^*M*^), the *fru *transcript that is sex-specifically spliced to produce FRU^M ^(*fru P1*), and each of three of the *fru *DNA-binding domains (f*ru*^*A*^, *fru*^*B*^, and *fru*^*C*^). Sequences for these oligonucleotides can be found in Additional file 11. All microarrays were printed in the laboratory of Dr. Eric Johnson at the University of Oregon (Eugene, OR) using slides coated with aldehyde chemistry and were postprocessed using the Nunc SuperChip Aldehyde protocol (Thermo Fisher Scientific, Waltham, MA).

All arrays were scanned using the GenePix 4100A scanner and GenePix Pro 5.0 software from Axon Instruments (Molecular Diagnostics, Sunnyvale, CA). Visual inspection of the microarray images filtered out florescence most likely not due to labeled cDNA binding; the data from these array elements was discarded. Array elements were only considered for further analysis if at least one channel (Cy3 or Cy5) had greater than 75% of the pixels with intensity values one standard deviation above background levels. All microarray normalization and statistical analyses were performed using the LIMMA package of BioConductor in the program R [16,59-61]. Global-loess normalization was used for all arrays, and significance was converted to *q *values using the *q *value application for R [13].

To determine the experimental reproducibility for the time course study, correlation values between replicates of each experiment were determined using Pearson's correlation on the logarithm of the ratio values for every oligonucleotide on the microarray. For each experiment, pair-wise Pearson correlation comparisons were performed on the three microarray replicates. All comparisons between replicates for the *tud *progeny experiments of the time course microarray study had correlations >0.75 and 27 of the 30 replicate comparisons had correlations >0.80. All comparisons between replicates for the wild type experiments in the time course microarray study had correlations >0.68 and 28 of the 30 replicate comparisons had correlations >0.80. See Additional file 4 for correlation values.

To identify the number of genes expressed during metamorphosis, we required a gene to have statistically analyzable expression data for at least one time point, in either of the sexes. See Additional file 4 for numbers of genes with expression during metamorphosis in males and females for both wild type (CS) pupae and *tud *progeny pupae.

Microarray data can be accessed at NIH GEO database with the following GEO accession number: GSE11316.

### Clustering of time course microarray expression data

Clusters were generated using the *tud *progeny data and the program Cluster [20]. Genes in the cluster must have had expression values for at least three of the five time points from both the *tud *progeny females and male datasets to be included in the cluster. The logarithms of the ratio values for each gene in each sex were median-centered and clustered. For clustering, we used Pearson correlation as the distance measure and defined similarity between clusters using average-linkage clustering. The cluster files outputted were imaged and analyzed using Java TreeView [62]. Co-regulated clusters were defined as any group of genes for which the average correlation was at least 0.80 and contained at least 15 members. All correlations between the clusters are <0.80 and only 31 of 703 pair-wise comparisons between clusters have correlations >0.70, demonstrating a high degree of separation between the clusters. In Additional file 6, the average expression for the genes in each cluster from the wild type male and female experiments is included; the data from each wild type sex is also median-centered separately, but was not used to mathematically generate these clusters.

The graphical images in Figure 3 were generated by averaging the logarithm of the ratio data for all member genes of each cluster at each time point. The cluster intensities represent the combined average for male and female *tud *progeny. Peaks and troughs for gene expression in each cluster were determined as follows: for each cluster, the mean of gene expression at all time points was calculated. A time point was considered to be at a peak if the average gene expression at a particular time point was greater than 2/3 of a standard deviation above the mean. Conversely, a time point was considered to be in a trough if the average gene expression at a particular time point was 2/3 of a standard deviation below the mean. To determine if the mean expression values of the declared peaks and troughs were significantly different than expected at random, a re-sampling analysis was conducted as follows: for each cluster, let *n *be the number of genes in the cluster. For each time point, *n *expression values were randomly selected with replacement from the list of all expression values from all time points in the cluster and the mean expression value was determined for each time point. This was repeated with 10,000 permutations and significance was declared if a peak or trough had a mean expression value greater than or less than, respectively, 99% of randomly permuted averages.

The dendrograms shown in Figure 5 were produced using the logarithms of the ratio data for every annotated gene spotted on the microarray, not including control spots. Clusters were generated using the program Cluster [20], Pearson correlation as the distance measure, and average-linkage clustering to define similarity between clusters. The outputted cluster files were then imaged using Java TreeView [62] and the resulting dendrograms were exported. Confidence values for each node were generated using the statistical package Pvclust in R [63]. Confidence values using the approximately unbiased *P *value were generated from multi-scale bootstrap resampling.

### Statistical analysis of overrepresented features

For all of the statistical analyses below, significance was declared if *P *< 0.05. Significant overrepresentations of functional annotations were generated with the program DAVID [22]. Unique GenBank accession number identifiers for the gene list and the whole set of unique GenBank accession numbers for all possible transcripts in the full array set (13,614 genes total) were used. When searching for overrepresented functional annotations using DAVID, the following categories were selected: all levels (or only Level 4) of each of the three Gene Ontology (GO) categories, Uniprot Sequence Features, Swiss-Prot Keywords, KEGG metabolic pathways, InterPro domains, PIR superfamily names, and SMART domains [64-69]. The gene ontology enrichment function in FlyMine, an integrated database for *Drosophila *and *Anopheles *genomics [23], was used as a secondary method to confirm the DAVID results. All functional categories presented in Figure 3 and all functional categories discussed in the text were found to be significantly overrepresented (*P *< 0.05) by FlyMine. Furthermore, the results from DAVID were additionally confirmed with FlyMine by identifying the ten most significant GO terms as determined by DAVID for each of the eight clusters described in detail in the text (a total of 80 functional terms). Sixty-four of these 80 significant functional groups (84%) identified by DAVID were also found to be significantly overrepresented (*P *< 0.05) by FlyMine.

Additionally, our own program was used to determine overrepresentation of chromosomal location and conserved genes in the clusters. This code used a gene's Flybase number as the unique identifier and the background set consisted of the whole set of unique Flybase numbers for all possible transcripts in the full array set (13558 genes total). Code is written in Java and is available upon request. Significance was declared using the binomial approximation of the hypergeometric test on the list of unique identifiers, with all possible unique identifiers in the full array set as background. Genes whose coding regions overlap with the 2,500 most conserved DNA elements, as generated by PhastCons [70] was determined. These highly conserved sequences range from hundreds to thousands of base pairs in length. In the previous study [70], multiple alignments of the genomic sequences of four insect species were used to determine conservation.

### Statistical analysis of differential expression during metamorphosis using F-statistics implemented in LIMMA

To identify genes with significant differential expression between the sexes or across time, a two-tiered approach using F-statistics was implemented (see Additional file 1 for more details). First, using contrasts in LIMMA [16], genes were identified with significant differences in expression according to time, sex, or the sex-time interaction (*q *< 0.15). Genes with significant differential expression in sex or the sex-time interaction were considered to be sex-biased, identifying 258 genes with sex-differential expression in the somatic tissues during metamorphosis. The 258 genes identified as having sex-differential expression according to the above criteria were further analyzed to determine at which stage(s) they show sex-differential expression. These analyses were conducted using moderated *t*-tests in LIMMA (*q *< 0.15) comparing the mean expression values of *tud *progeny males and females at each of the five time points for which expression data was generated (0, 24, 48, 72, and 96 hour APF).

To identify genes that are expressed in the male and female germlines during metamorphosis, a similar approach was implemented (see Additional file 1 for more details). First, genes were identified with male- or female-biased expression in the wild type tissues using LIMMA contrasts with sex as the independent factor (*q *< 0.15). We expect genes with germline expression to be highly expressed, and as such we required genes with significant differential expression to also have at least a 1.2 fold difference in expression between the sexes. To identify genes expressed in the male germline, a LIMMA contrast analysis on the wild type male and male *tud *progeny data was performed, with genotype as the independent factor. Genes expressed in the male germline were those male-biased genes that additionally had significantly greater expression in the wild type males than *tud *progeny males (genotype as independent factor;*q *< 0.15). These genes were additionally required to show at least a 1.2 fold increase in expression in wild type males as compared to *tud *progeny males. In addition, to avoid false negatives, genes were included that are expressed in wild type males for at least four of the five time points examined and which have no expression data in the wild type female and male *tud *progeny experiments. The set of genes with expression in the female germline was defined in a similar manner, using the female wild type and *tud *progeny expression data.

### Statistical identification of genes expressed downstream of the sex-determination hierarchy

To identify genes with sex-differential expression in the somatic tissues downstream of the sex hierarchy, genes with significant expression differences between male and females flies were first identified (the CS and Berlin experimental data was analyzed together; *q *< 0.15). The list was refined by requiring a gene to also be significantly, differentially expressed (*q *< 0.15; one tailed *t*-test) between male and female *tud *progeny (sex-differential expression was required to be in the same direction for the wild type and *tud *progeny experiments). The list was further refined to identify genes regulated downstream of *tra*, by identifying genes that were differentially expressed (*q *< 0.15; one-tailed *t*-test) between females and *tra *pseudomales. Here, it was required that genes with female-biased expression have higher expression in wild type females and genes with male-biased expression have higher expression in *tra *pseudomales.

Finally, genes expressed downstream of *dsx *in somatic tissues, were identified by refining the list of genes expressed downstream of *tra*, and requiring a gene to be significantly differentially expressed (*q *< 0.15; one-tailed *t*-test) between females and *dsx*^*D *^pseudomales. Here it is assumed that genes with female-biased expression have higher expression in wild type females and genes with male-biased expression have higher expression in *dsx*^*D *^pseudomales. To avoid false negatives in the identification of genes regulated downstream of *dsx*, which might have resulted from genes that failed to be significantly, differentially expressed in the *tra *pseudomale comparison, the DSX-regulated gene set was extended to include genes that were differentially expressed between wild type males and females (CS and Berlin; *q *< 0.15), *tud *males and females (*q *< 0.15), and female and *dsx*^*D *^pseudomales at a more stringent statistical threshold (*q *< 0.05), thus excluding the requirement that the gene was differentially expressed in the *tra *experiments. To examine DSX modes of regulation, the genes regulated downstream of *dsx *were examined that displayed significant differential expression (*q *< 0.15) in either of the *dsx *null comparisons.

### Identification of DSX binding sites in regulatory sequences

To identify the presence of DSX binding sites in the regulatory regions of Drosophila genes, we used the known DSX binding site (ACAAWGT) [46]. We searched for the presence of two DSX binding sites in a region containing 2000 base pairs upstream from the transcription start and the first intron for each gene. In total, 13,449 genes were included in the search; the genomic sequence was determined from Release 4.0 of the Drosophila genome. Overrepresentation of genes with the DSX-binding was determined using a hypergeometric test compared against all genes in the Drosophila genome.

## Abbreviations

APF: (after puparium formation); CS: (Canton-S); DAVID: (Database for Annotation, Visualization and Integrated Discovery); *dsx*: (*doublesex*); FC: (fold change); *fru*: (*fruitless*); LIMMA: (Linear Models for Microarray Data); *tra*: (*transformer*); *tud*: (*tudor*); wpp: (white pre-pupal).

## Authors' contributions

All of the authors conceived and designed the experiments, analyzed the data, contributed reagents/materials/analysis tools, and wrote the paper. MSL and LES performed the experiments. All authors read and approved the final manuscript.

## Supplementary Material

Additional file 1 **Details of F-statistic analyses for time course microarray data.** This document details the F-statistics and contrast matrix designs for identifying sex-differentially expressed genes and genes expressed in the male and female germlines using LIMMA. These analyses were conducted on the time course gene expression data. For this data, probes from wild type males, wild type females, *tud *progeny males, and *tud *progeny females were all separately hybridized against a common reference sample at five time points during metamorphosis (0, 24, 48, 72, and 96 hour APF). Click here for file

Additional file 2 **Numbers of genes identified in the time course experiment by F-statistic analyses implemented in LIMMA.** This table reports the number of genes with significant differences in transcript abundance (q < 0.15), as identified using LIMMA contrasts analyses on the expression data from wild type or *tud *progeny males and females. For more details on contrast matrix designs, see Additional file 1. Click here for file

Additional file 3 **Genes with somatic, sex-differential expression across metamorphosis and at 0, 24, 48, 72, and 96 hour APF stages during metamorphosis.** The first tab lists the genes identified as being sex-differentially expressed during metamorphosis as identified by F-statistic analyses implemented in LIMMA, using the time course microarray data. The second through sixth tabs lists the genes identified as sex-differentially expressed for each of the five time points examined (0, 24, 48, 72, and 96 hours APF). The genes in tabs two through six are all a subset of the genes listed in the first tab. Click here for file

Additional file 4 **Correlations of data from array experiments and number of genes identified in the microarray experiments.** This first tab contains Pearson correlations between microarray replicates and the number of genes expressed for the time course microarray experiments. This second tab contains Pearson correlations between microarray replicates and the number of genes expressed when microarray comparisons between sex determination hierarchy mutants and wild type animals were performed. Click here for file

Additional file 5 **Gene lists of 38 clusters from time course microarray experiments.** Lists of genes from each of the 38 clusters identified and presented in Figure 3 and Additional file 6. Clusters were generated using gene expression data from male and female *tud *progeny at five time points during metamorphosis (0, 24, 48, 72, and 96 hours APF). The gene list from each cluster is presented in the individual tabs. Click here for file

Additional file 6 **Average gene expression of the 38 clusters identified from the time course microarray experiments and presented in Figure 3.** Clusters were generated using gene expression data from male and female *tud *progeny at five time points during metamorphosis. The wild type data is included, but has no weight in the cluster formation. For each cluster, the abscissa indicates the five time points during metamorphosis examined (0, 24, 48, 72, and 96 hour APF) and the ordinate indicates the average expression value for each genotype examined. Expression profiles for each cluster were generated by averaging the gene expression data at each time point for every gene in the cluster, in both sexes. Average expression values are represented by teal for wild type males, blue for wild type females, green for *tud *males, and red for *tud *females. Gene lists can be found in Additional file 5. Functional categories that were overrepresented among the genes in the cluster, as determined by the program DAVID (*P *< 0.05 [22]), are described in Additional file 7. Click here for file

Additional file 7 **DAVID analysis of 38 clusters from time course microarray experiments.** Results of functional analysis from the program DAVID for each of the 38 clusters identified and presented in Figure 3 and Additional files 5 and 6. Clusters were generated using gene expression data from male and female *tud *progeny at five time points during metamorphosis (0, 24, 48, 72, and 96 hours APF). The DAVID functional analysis of each cluster is presented on individual tabs. Click here for file

Additional file 8 **Genes that are expressed in the male germline during metamorphosis.** This table lists the genes identified as being expressed in or as a consequence of the male germline. Also included in the second tab are over-represented functional categories as determined by DAVID. Click here for file

Additional file 9 **Genes that are expressed in the female germline during metamorphosis.** This table lists the genes identified as being expressed in or as a consequence of the female germline. Also included in the second tab are over-represented functional categories as determined by DAVID. Click here for file

Additional file 10 **Genes that show somatic, sex-differential expression at 48 hour APF, and genes expressed downstream of *tra *and *dsx *at 48 hour APF.** The first tab lists genes with somatic, sex-differential transcript abundance differences at 48 hour APF during metamorphosis, the second tab lists genes with somatic sex differential transcript abundance downstream of *tra *at 48 hour APF during metamorphosis, and the third tab lists genes with somatic, sex-differential transcript abundance downstream of *dsx *at 48 hour APF during metamorphosis. Click here for file

Additional file 11 **Oligonucleotide sequences used as controls on the microarrays for genes in the Drosophila sex determination hierarchy.** This table includes the sequence information for the control microarray spots for genes in the sex determination hierarchy. Included are control spots for *Sex lethal *(*Sxl*), *transformer *(*tra*), the female-specific region of *doublesex *transcripts (*dsx*^*F*^), the male-specific region of *doublesex *transcripts (*dsx*^*M*^), the PI-transcript of *fruitless *(*fru P1*), the A, B and C DNA binding domain of *fruitless *(*fru*^*A*^, *fru*^*B*^, *fru*^*C*^). Click here for file
