# Supplementary material for: Somatic, germline and sex hierarchy regulated gene expression during Drosophila metamorphosis
Source: BMC Genomics. 2009 Feb 13;10:80. doi: 10.1186/1471-2164-10-80 (PMC2656526; doi:10.1186/1471-2164-10-80)
Supplement: Additional file 1 — Details of F-statistic analyses for time course microarray data. This document details the F-statistics and contrast matrix designs for identifying sex-differentially expressed genes and genes expressed in the male and female germlines using LIMMA. These analyses were conducted on the time course gene expression data. For this data, probes from wild type males, wild type females, tud progeny males, and tud progeny females were all separately hybridized against a common reference sample at five time points during metamorphosis (0, 24, 48, 72, and 96 hour APF). [file 1471-2164-10-80-S1.pdf]

This document details the F-statistics and contrast matrix designs for identifying sex-differentially expressed genes and genes expressed in the male and female germlines using LIMMA [1-4]. These analyses were conducted on the time course gene expression data. For this data, wild type males, wild type females, *tud* progeny males, and *tud* progeny females were all separately hybridized against a common reference sample at five time points during metamorphosis (0, 24, 48, 72, and 96 hour APF) with three microarray replicates.

Statistical analyses of microarray data

All arrays were scanned using the GenePix 4100A scanner and GenePix Pro 5.0 software from Axon Instruments (Molecular Diagnostics, Sunnyvale, CA). Visual inspection of the microarray images filtered out florescence most likely not due to labeled cDNA binding; the data from these array elements was flagged as absent. Array elements were only considered for further analysis if at least one channel (Cy3 or Cy5) had greater than 75% of the pixels with intensity values one standard deviation above background levels (columns “B532+1SD” and “B635+1SD” in gpr file, respectively) and if they were not flagged by either GenePix Pro or visual inspection. All microarray normalization and statistical analyses were performed using the LIMMA package of BioConductor in the program R [1-4]. Global-loess normalization was used for all arrays, and significance was converted to *q* values using the *q* value application for R [5]. In the analyses, the design matrix for the analyses labeled the microarray experiments in the following manner: wild type males from 0, 24, 48, 72, and 96 hour APF were labeled as wtM_0hr, wtM_24hr, wtM_48hr, wtM_72hr, and wtM_96hr, respectively. Similar labeling was used for wild type females, *tud* progeny males, and *tud* progeny females.

Identification of sex-differentially expressed genes in the somatic tissues

To identify genes with somatic, sex-differential expression, F-statistics were implemented using contrasts in LIMMA. First contrast matrices for the appropriate analysis were created and then fitted to the model. The *P* values for the resulting F-statistic were then converted into *q* values. Genes with significant expression differences according to the sex factor were determined by the following contrast design: [FvM0=tudF_0hr-tudM_0hr, FvM24=tudF_24hr-tudM_24hr, FvM48=tudF_48hr-tudM_48hr, FvM72=tudF_72hr-tudM_72hr, FvM96=tudF_96hr-tudM_96hr]. Genes with significant expression differences according to the sex-time interaction were determined by the following contrast design: [FvM_24v0=(tudF_24hr-tudF_0hr)-(tudM_24hr-tudM_0hr), FvM_48v0=(tudF_48hr-tudF_0hr)-(tudM_48hr-tudM_0hr), FvM_72v0=(tudF_72hr-tudF_0hr)-(tudM_72hr-tudM_0hr), FvM_96v0=(tudF_96hr-tudF_0hr)-(tudM_96hr-tudM_0hr), FvM_48v24=(tudF_48hr-tudF_24hr)-(tudM_48hr-tudM_24hr), FvM_72v24=(tudF_72hr-tudF_24hr)-(tudM_72hr-tudM_24hr), FvM_96v24=(tudF_96hr-tudF_24hr)-(tudM_96hr-tudM_24hr), FvM_72v48=(tudF_72hr-tudF_48hr)-(tudM_72hr-tudM_48hr), FvM_96v48=(tudF_96hr-tudF_48hr)-(tudM_96hr-tudM_48hr), FvM_96v72=(tudF_96hr-tudF_72hr)-(tudM_96hr-tudM_72hr)]. Genes were declared to have

sex-differential expression in the somatic tissues if expression values were determined to change significantly according to either the sex or sex-time interaction terms ($q<0.15$).

Two-hundred-fifty-eight genes were thus identified as showing sex-differential expression in the somatic tissues during metamorphosis. To determine how these genes were sex-differentially expressed at the five time points examined in this study (0, 24, 48, 72, and 96 hour APF), moderated *t*-tests were performed in R comparing the mean expression in *tud* progeny males and females at each time point separately. The resulting *P* values of the 258 genes were converted to *q* values using the *q* value application for R [5], and significance was declared at a $q<0.15$ level.

Identification of genes expressed in the male or female germlines

To identify genes with expression differences due to the presence of a germline, F-statistics were also implemented using contrasts in LIMMA. Again, contrast matrices for the appropriate analysis were created and then fitted to the model. The *P* values for the resulting F-statistic were then converted into *q* values. Genes were first identified with sex-differential expression between wild type males and females using the following contrast design: [FvM0=wtF_0hr-wtM_0hr, FvM24=wtF_24hr-wtM_24hr, FvM48=wtF_48hr-wtM_48hr, FvM72=wtF_72hr-wtM_72hr, FvM96=wtF_96hr-wtM_96hr]. Genes with significant ($q<0.15$) and at least a 1.2 fold-change expression differences between the sexes were kept, leaving 3194 genes to be analyzed (2320 and 883 female- and male-biased genes, respectively; nine genes have one male-biased isoform and one female-biased isoform).

To identify genes expressed in the male germline, genes with significant expression differences in males according to the genotype factor (wild type vs. *tud* progeny) were determined by the following contrast design: [wtVtud0hr=wtM_0hr-tudM_0hr, wtVtud24hr=wtM_24hr-tudM_24hr, wtVtud48hr=wtM_48hr-tudM_48hr, wtVtud72hr=wtM_72hr-tudM_72hr, wtVtud96hr=wtM_96hr-tudM_96hr]. Male-biased genes (of the 883 found above) with significant ($q<0.15$) and at least a 1.2 fold-change higher expression in wild type males as compared to *tud* progeny males were declared as being expressed in the male germline (586 genes). In addition, to avoid false negatives, genes were included in the male germline that were expressed in at least four time points in wild type males and that had no expression in wild type females and *tud* progeny males at all five time points examined in this study. Seventy-three additional genes were thus included. See Additional file 8 for list of the 659 genes expressed in or as a consequence of the male germline.

To identify genes expressed in the female germline, genes with significant expression differences in females according to the genotype factor (wild type vs. *tud* progeny) were determined by the following contrast design: [wtVtud0hr=wtF_0hr-tudF_0hr, wtVtud24hr=wtF_24hr-tudF_24hr, wtVtud48hr=wtF_48hr-tudF_48hr, wtVtud72hr=wtF_72hr-tudF_72hr, wtVtud96hr=wtF_96hr-tudF_96hr]. Female-biased genes (of the 2320 found above) with significant ($q<0.15$) and at least a 1.2 fold-change higher expression in wild type females as compared to *tud* progeny females were declared as being expressed in the female germline (342

genes). In addition, to avoid false negatives, genes were included in the female germline that were expressed in at least four time points in wild type females and that had no expression in wild type males and *tud* progeny females at all five time points examined in this study. No such genes were identified for the female germline. See Additional file 9 for list of the 342 genes expressed in or as a consequence of the female germline.

References

1. Smyth GK: **Linear models and empirical bayes methods for assessing differential expression in microarray experiments**. *Statistical applications in genetics and molecular biology* 2004, **3**:Article3.
2. Smyth GK: **Limma: linear models for microarray data**. In: *Bioinformatics and Computational Biology Solutions using R and Bioconductor*. Edited by R. Gentleman VC, S. Dudoit, R. Irizarry, W. Huber. New York: Springer; 2005: 397-420.
3. Smyth GK, Speed T: **Normalization of cDNA microarray data**. *Methods* 2003, **31**(4):265-273.
4. Gentleman RC, Carey VJ, Bates DM, Bolstad B, Dettling M, Dudoit S, Ellis B, Gautier L, Ge Y, Gentry J *et al*: **Bioconductor: open software development for computational biology and bioinformatics**. *Genome biology* 2004, **5**(10):R80.
5. Storey JD: **The positive flase discovery rate: A Bayesian interpretation and the q-value**. *Annals of Statistics* 2003(31):2013-2035.
