# Supplementary figures and images for: Somatic, germline and sex hierarchy regulated gene expression during Drosophila metamorphosis
Source: BMC Genomics. 2009 Feb 13;10:80. doi: 10.1186/1471-2164-10-80 (PMC2656526; doi:10.1186/1471-2164-10-80)

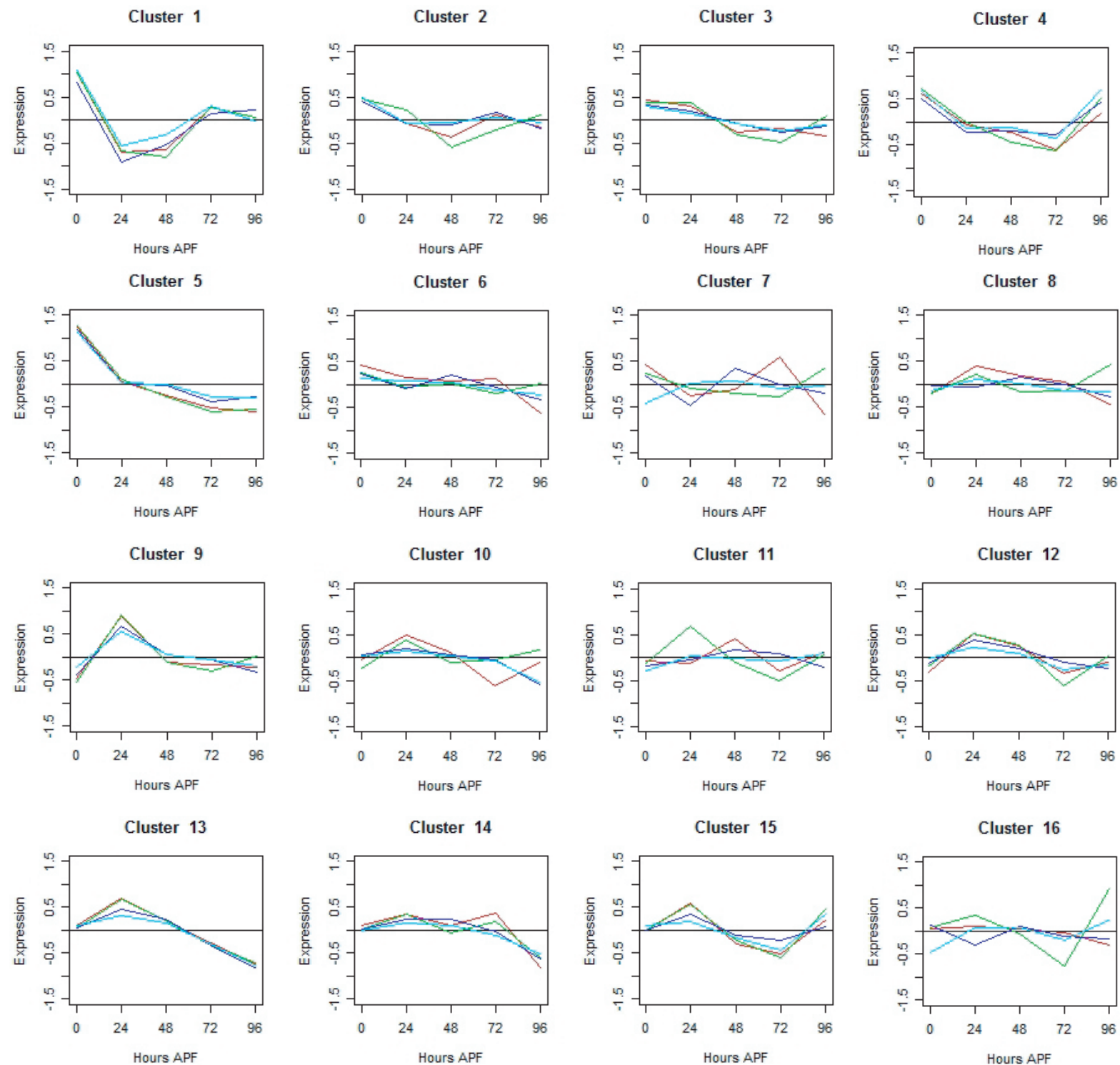

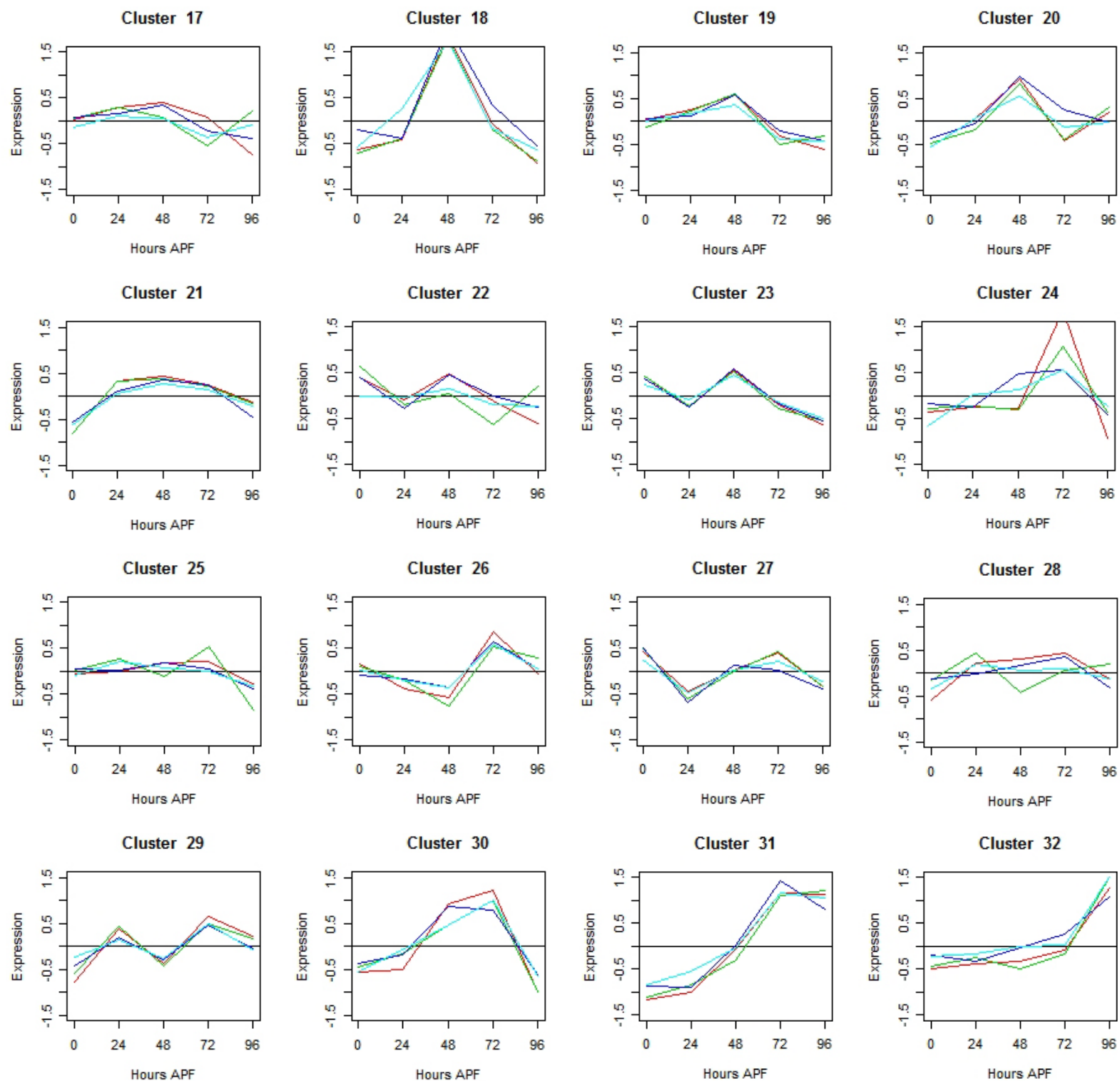

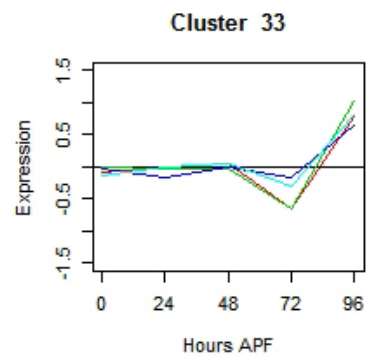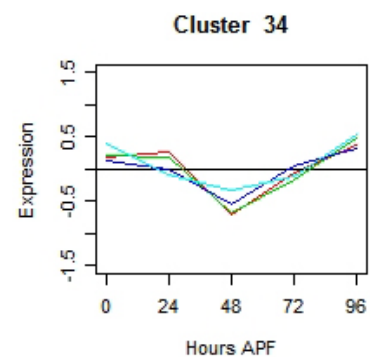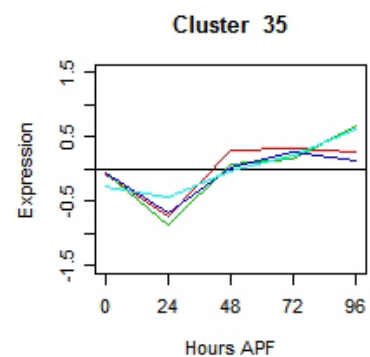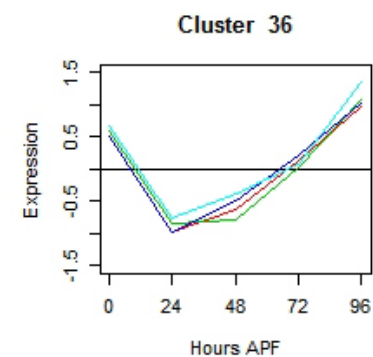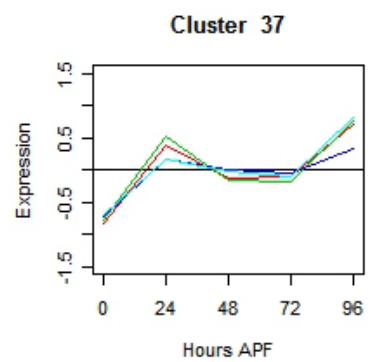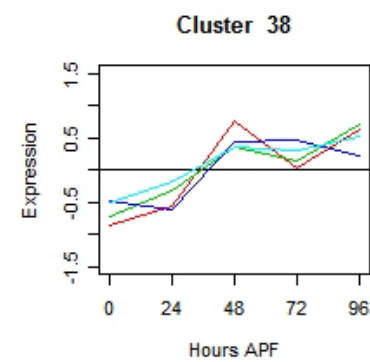

Supplement: Additional file 6 — Average gene expression of the 38 clusters identified from the time course microarray experiments and presented in Figure 3. Clusters were generated using gene expression data from male and female tud progeny at five time points during metamorphosis. The wild type data is included, but has no weight in the cluster formation. For each cluster, the abscissa indicates the five time points during metamorphosis examined (0, 24, 48, 72, and 96 hour APF) and the ordinate indicates the average expression value for each genotype examined. Expression profiles for each cluster were generated by averaging the gene expression data at each time point for every gene in the cluster, in both sexes. Average expression values are represented by teal for wild type males, blue for wild type females, green for tud males, and red for tud females. Gene lists can be found in Additional file 5. Functional categories that were overrepresented among the genes in the cluster, as determined by the program DAVID (P < 0.05 [22]), are described in Additional file 7. [file 1471-2164-10-80-S6.pdf]
